# Design and modeling of area-enhanced, bi-porous, evaporative macrochannel for building facade cooling

**DOI:** 10.1038/s44435-026-00021-6

**Published:** 2026-10-07

**Authors:** Gautier Rouaze, Zhengmao Lu

**Affiliations:** EPFL, Lausanne, Switzerland

**Keywords:** Energy science and technology, Engineering, Mathematics and computing

## Abstract

Facade-integrated evaporative cooling offers a pathway to high heat rejection for buildings with limited rooftop area. However, it needs to operate under fixed footprint and parasitic power constraints, requiring surface enhancement strategies that enable more efficient heat removal. We develop a fan-power-constrained conjugate heat-mass transfer framework to evaluate facade-integrable evaporative macrochannels. We show that hydrogel-only pin fins enhance evaporation into channels but provide little increase in base heat removal due to high internal thermal conduction resistance. To overcome this bottleneck, we introduce a Hydrogel Overlayer on Metal Scaffold (HOMeS) architecture that separates heat spreading and capillary wetting across pore scales. Bayesian optimization identifies near-optimal pin-fin geometries, which, for a 200 × 200 mm panel at a fan power density of 125 Wm^−2^, deliver ~7 × higher base heat removal than a flat evaporating channel. We demonstrate that evaporative texturing provides significant performance improvement only when thermal coupling to the interface is preserved.

## Introduction

The continued expansion of cloud computing and data-intensive services is driving a rapid growth in datacenter electricity consumption, with thermal management representing a major fraction of that demand^1–3^. Reducing the effective heat-sink temperature without incurring large parasitic power penalties therefore presents a central challenge in thermal science and engineering.

At the building scale, heat rejection is increasingly constrained by footprint availability and architectural integration. Rooftop real estate is limited and often prioritized for photovoltaics, while ground-level installations are restricted by space and infrastructure. Building facades offer a unique opportunity: they can, in principle, provide large cooling area but impose strict constraints on thickness, orientation, and auxiliary power.

Radiative cooling has attracted significant attention because it can achieve sub-ambient temperatures with very low operating energy^4,5^. However, the net cooling flux is typically limited to ~100 Wm^−2^ and degrades strongly for vertical or tilted orientations due to reduced sky-view factors and increased infrared exchange with the surrounding built environment^6,7^. Although recent angular-selective emitters partially mitigate these effects^8–10^, radiative facade cooling remains fundamentally constrained by geometry and orientation.

Evaporative cooling provides a different technological pathway by exploiting the large latent heat of vaporization. Facade-scale evaporative concepts, including water-soaked porous walls^11,12^, spray-assisted double-skin facades^13–15^, and evaporative textile skins^16^, can deliver substantially higher heat rejection when wet-bulb depression is favorable. A common challenge across these approaches is maintaining a uniformly wetted, evaporation-active surface under sustained operation on vertical or inclined facades. Conventional porous or fibrous media, including cellulose pads, textiles, and porous ceramics^13,16–19^ typically require substantial thickness to reliably retain and distribute water. In practice, this increases hydraulic resistance to the evaporating interface during continuous operation. Moreover, the significant solid fraction of these materials limits the effective liquid-air interfacial area available for evaporation.

Hydrogels have therefore recently attracted interest as alternative wicking layers because they store water within a continuous polymer network and can sustain a large effective liquid-air interfacial area for evaporation even when implemented as thin, conformal layers. Their fine pore structure can generate large capillary pumping pressures, while their synthesis versatility allows conformal integration with a wide range of substrates^20,21^. Previously, hydrogel systems have been explored for passive thermal regulation, which can be applied in buildings, photovoltaic modules, and wearable or electronic devices^22–27^. However, hydrogel layers are commonly designed to be thick to increase storage capacity, which introduces a large internal thermal resistance and limits the operational heat flux^21,28^.

This limitation is fundamentally linked to the low thermal conductivity (*k*) of hydrogels, which is close to that of water: when the heat source is separated from the evaporating interface by a thick, low-*k* medium, conduction becomes the dominant bottleneck. To reduce the conduction penalty, recent studies have coated hydrogel onto copper pin-fins^27^ or infused it into copper foams^29,30^ to improve thermal coupling between the heat source and the evaporating surface. However, the performance of these systems at high cooling fluxes remains limited. In particular, the surface-area enhancement effect under forced convection and the liquid supply pathways capable of sustaining uniform wetting at high evaporation rates have not been systematically addressed. On the other hand, achieving high-performance facade-integrated evaporative cooling suitable for high-thermal-load buildings requires designs that explicitly account for these coupled constraints while operating under realistic parasitic-power limits.

**Fig. 1:**
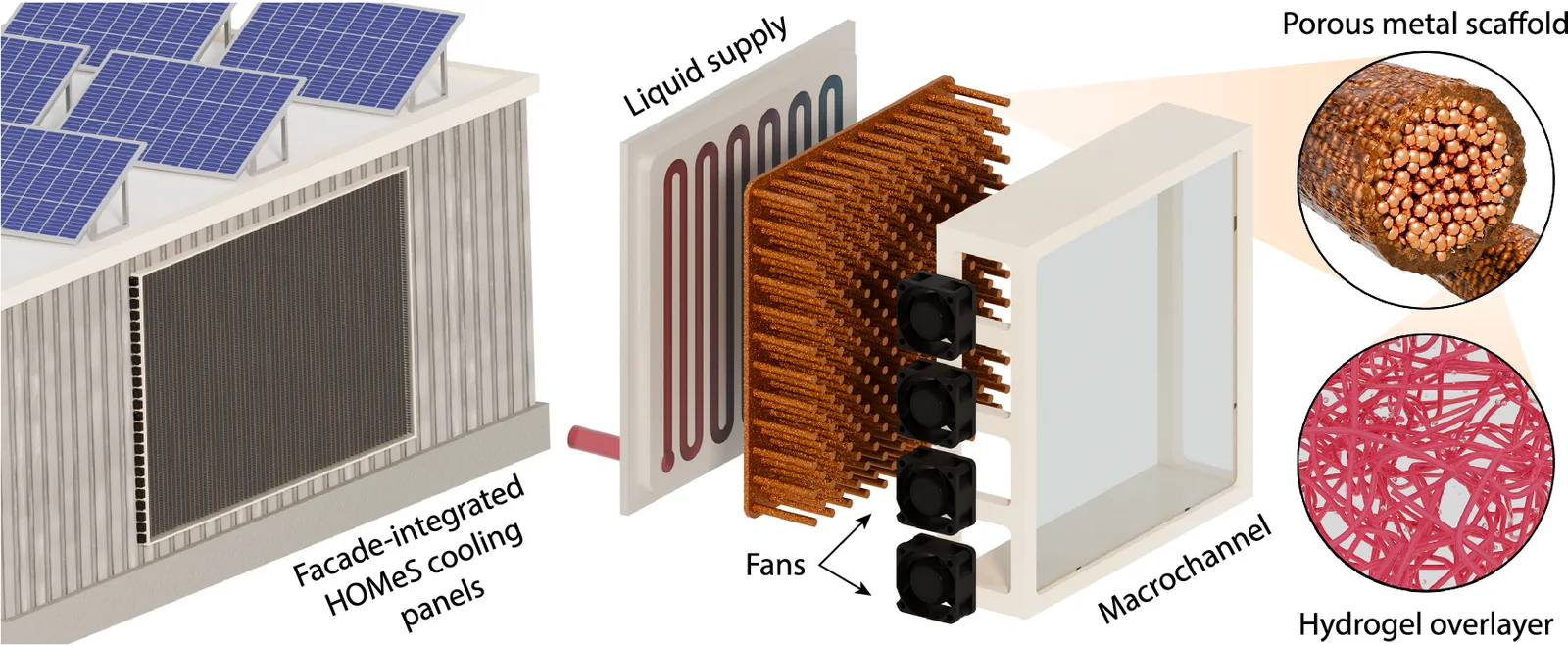
Concept of facade-integrated HOMeS evaporative cooling panels. Left: a large-area evaporative macrochannel module mounted on a building facade (with rooftop space prioritized for photovoltaics). Center: exploded view of a single panel showing the liquid-supply manifold, a pin-fin-textured evaporative macrochannel, and compact fans that drive airflow through the channel. Right: the bi-porous Hydrogel Overlayer on Metal Scaffold (HOMeS) interface, in which a liquid-saturated porous metal scaffold provides a conductive heat-spreading network and permeable liquid-distribution pathways, while a thin hydrogel overlayer maintains a continuous wetted liquid-air interface.

Motivated by these thermal and hydraulic considerations, here, we propose a Hydrogel Overlayer on Metal Scaffold (HOMeS) architecture, consisting of a thin hydrogel skin integrated with a porous metallic support, inherently forming a bi-porous design (Fig. 1). At the air-facing surface, a continuous hydrogel overlayer forms a fully wetted evaporation interface. This layer provides capillary suction to draw liquid from the interior and ensures a spatially uniform evaporation area. The total thickness of the hydrogel layer is tightly constrained: it must be thick enough to fully cover the underlying metal scaffold and prevent dry exposure, yet remain as thin as possible to minimize added thermal resistance and vapor-side diffusion losses.

Beneath this hydrogel skin, a highly permeable porous-metal scaffold serves as the primary load-bearing and heat-spreading element. The metallic framework provides high effective thermal conductivity for lateral heat redistribution and low hydraulic resistance for liquid transport across the fixed footprint. Part of the hydrogel phase is interlocked with the scaffold pores to anchor the surface overlayer, ensure mechanical integrity, and maintain leak-free attachment under sustained wetting and evaporation.

We develop a numerical framework to assist us in designing and optimizing the HOMeS structure into pin-fin arrays integrated with evaporative macrochannel panels. The baseline architecture considered here is a confined evaporative macrochannel (a thin module comprising an air passage with characteristic hydraulic dimensions on the order of tens of millimeters) in which a continuously wetted internal surface evaporates and cools a panel base under a fan-power constraint. Liquid transport within the system is not explicitly resolved; the feasibility for sustained wetting is evaluated separately based on the pressure balance between viscous loss and capillary pumping given the predicted evaporation fluxes. Under steady liquid supply, we use coupled conjugate heat and mass-transfer simulations to quantify base heat removal under fixed footprint and fan-power constraints and identify regimes in which surface texturing provides net performance gains in panel-relevant performance rather than merely enhanced air-side cooling. By combining this physics-based framework with Bayesian optimization, we showcase an efficient navigation of the pin-fin design space and uncover near-optimal geometries without exhaustive parametric sweeps, establishing actionable design rules for high-flux, facade-integrated evaporative cooling panels.

## Results

**Fig. 2:**
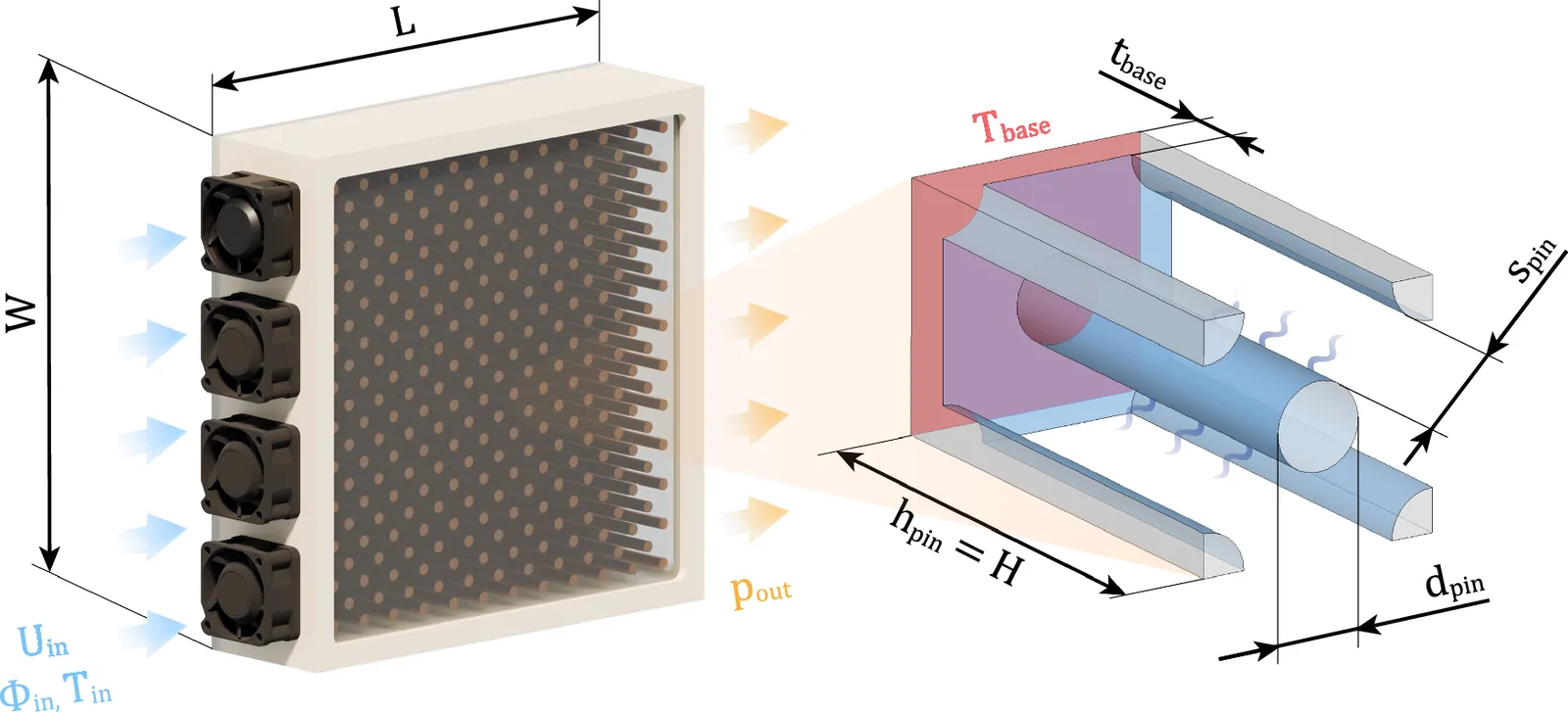
Schematic of the confined evaporative panel considered in the simulations. Air enters from the left plenum (velocity *U*_in_, temperature *T*_in_ = 25 °C, relative humidity *ϕ*_in_ = 50%) and exits on the right side at zero gauge pressure *p*_out_ = 0 Pa. The base plate is held isothermal at *T*_base_ = 35 °C. Baseline dimensions are width *W* = 200 mm, flow length *L* = 200 mm, macrochannel gap *H* = 25 mm, and base thickness *t*_base_ = 2 mm. For area-enhanced cases, cylindrical pin-fins of diameters *d*_pin_ = 2 mm, spacing *s*_pin_ = 5 mm and height *h*_pin_ = *H* are used, yielding an interface-to-footprint area ratio *A*_interface_/*A*_footprint_ ≈ 4.

A reduced-domain conjugate numerical model resolves coupled momentum, energy, and water-vapor transport in the air channel together with heat conduction through the solid regions (governing equations and boundary conditions are given in the *Methods* section). The baseline macrochannel has width *W* = 200 mm, streamwise length *L* = 200 mm, and gap height *H* = 25 mm (Fig. 2). The imposed base temperature *T*_base_ is the isothermal temperature of the panel base plate and represents the coupling to an external liquid-side loop rather than the indoor air temperature. A value of 35 °C is representative of the condenser side of a vapor-compression cycle whose heat the panel rejects to the ambient. Lower setpoints, for which the panel directly supplies a cooled liquid stream for space or process cooling, are also possible. The appropriate *T*_base_ is therefore a design choice set by the cooling load and the downstream system, and its effect is quantified by the base-temperature sensitivity in Supplementary Note S2. For this study, *T*_base_ is fixed at 35 °C, while the inlet air is prescribed at *T*_in_ = 25 °C and relative humidity *ϕ*_in_ = 50% at 1 atm. For area-enhanced cases, cylindrical pin fins are arranged on a square lattice rotated 45° relative to the mean flow; the baseline enhanced surface uses pin diameter *d*_pin_ = 2 mm and spacing *s*_pin_ = 5 mm with height *h*_pin_ = *H*, yielding the ratio between water-air interface area and footprint area *A*_interface_/*A*_footprint_ ≈ 4. Here, *s*_pin_ denotes the edge-to-edge spacing between adjacent pins (so the center-to-center pitch is *p* = *d*_pin_ + *s*_pin_).

Given that facade-integrated panels are often constrained in terms of footprint and thickness, typically fixed by architectural integration, performance cannot be compared meaningfully by prescribing an inlet velocity, because different surface textures impose different pressure drops and would therefore operate at different parasitic power. Instead, to enable a fair comparison across geometries, the operating point is defined by a specified fan power density *q*_fan_. For each geometry and operating condition, the corresponding mean inlet velocity is determined implicitly by solving the momentum balance, including intake and exhaust losses (see *Methods* section). Evaporation at the liquid–air interface is modeled assuming a fully wetted surface with the interfacial vapor mass fraction fixed at saturation at the local interface temperature. Capillary pumping feasibility toward the evaporating surface is addressed for the bi-porous structure in Supplementary Note S1.

### Baseline flat macrochannels

**Fig. 3:**
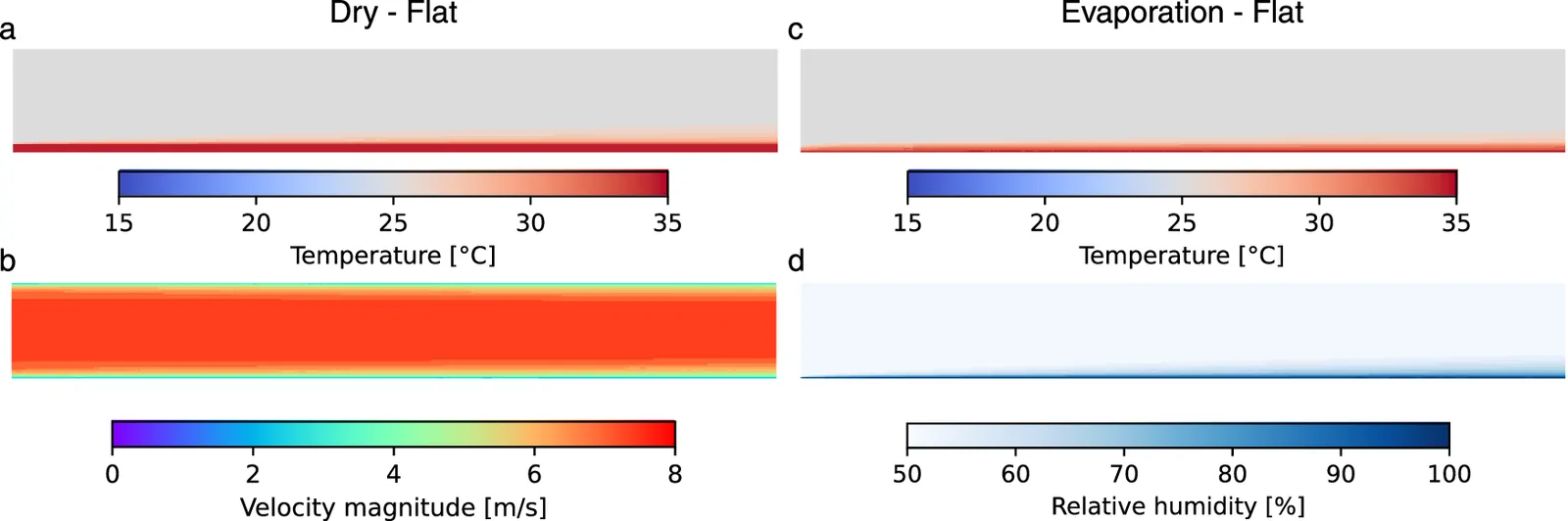
Flat macrochannel, dry versus evaporative convection at a fan power density of 125 Wm^−2^. Contour map from the two-dimensional simulation in the length-height plane for **a** dry convection case, temperature, **b** dry convection case, velocity magnitude, **c** evaporating hydrogel case, temperature, and **d** evaporating hydrogel case, relative humidity. Flow is left to right, and the base is held isothermal at 35 °C. In both cases, the transport boundary layer remains thin along the macrochannel: in **a** the large near-wall temperature gradient sustains high sensible heat transfer, in **c** interfacial cooling reduces it, and in **d** the thin species boundary layer produces a high interfacial mass fraction gradient and thus high latent heat extraction.

As a baseline, convective heat transfer over a dry flat macrochannel is compared with an identical channel in which the base is replaced by a fully wetted hydrogel layer, without pin fins. The active surface consists of a flat layer of thickness *t*_base_ = 2 mm, modeled either as copper with *k*_base_ = 400 Wm^−1^ K^−1^ (dry case) or as hydrogel with *k*_hyd_ = 0.6 Wm^−1^ K^−1^ (evaporating case). Figure 3a, b show temperature and velocity fields from the two-dimensional length–height simulation for the dry case at *q*_fan_ = 125 Wm^−2^. The thermal boundary layer develops along the channel but remains thin over most of the length at this fan power, consistent with the low pressure drop of the unobstructed macrochannel. Figure 3c, d present temperature and relative humidity fields for the corresponding evaporating flat hydrogel case. The vapor mass diffusion boundary layer is similarly thin, producing large interfacial gradients of vapor mass fraction and strong latent heat extraction.

**Fig. 4:**
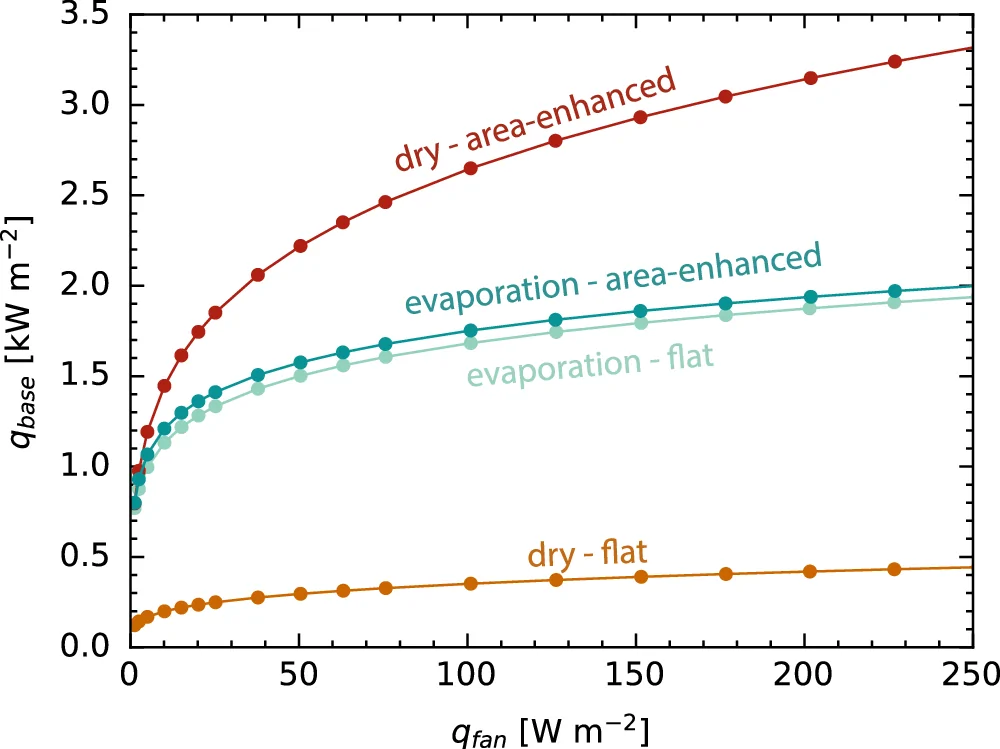
Comparison of the dissipated base heat flux *q*_base_ as a function of the fan power density *q*_fan_ for four cases. *dry-flat* (orange), *dry-area-enhanced* (red), *evaporation-flat* (light green), *evaporation-area-enhanced* (dark green). At matched fan power, evaporation on a flat hydrogel provides a large latent-heat boost over the dry flat channel (light green vs orange, ≈ 5 times at 125 Wm^−2^), whereas adding pin-fins under evaporation yields only a slight additional gain (dark green vs light green).

Because the wetted area equals the footprint and the residence time is short for the operating point shown in Fig. 3d, the bulk relative humidity rise remains modest (the average outlet relative humidity *ϕ*_out_ is about 4 percentage points higher than *ϕ*_in_ at *q*_fan_ = 125 Wm^−2^). Even in this modest-humidification regime, evaporation provides a large enhancement in the base cooling power: Fig. 4 shows that at matched fan power, the flat evaporating configuration (light green) removes approximately five times more heat than the dry flat channel (orange) (at 125 Wm^−2^). For the fixed footprint and channel length considered here, the flat evaporating case exhibits diminishing returns at high fan powers, reflecting the combined effects of reduced residence time and streamwise reduction in vapor mass transfer driving force as the air warms and humidifies. This motivates (i) boundary-layer mixing strategies that raise local transfer coefficients upstream where driving forces remain high and (ii) geometric redesigns that mitigate premature approach to saturation in the downstream portion of the channel.

### Area enhancement

**Fig. 5:**
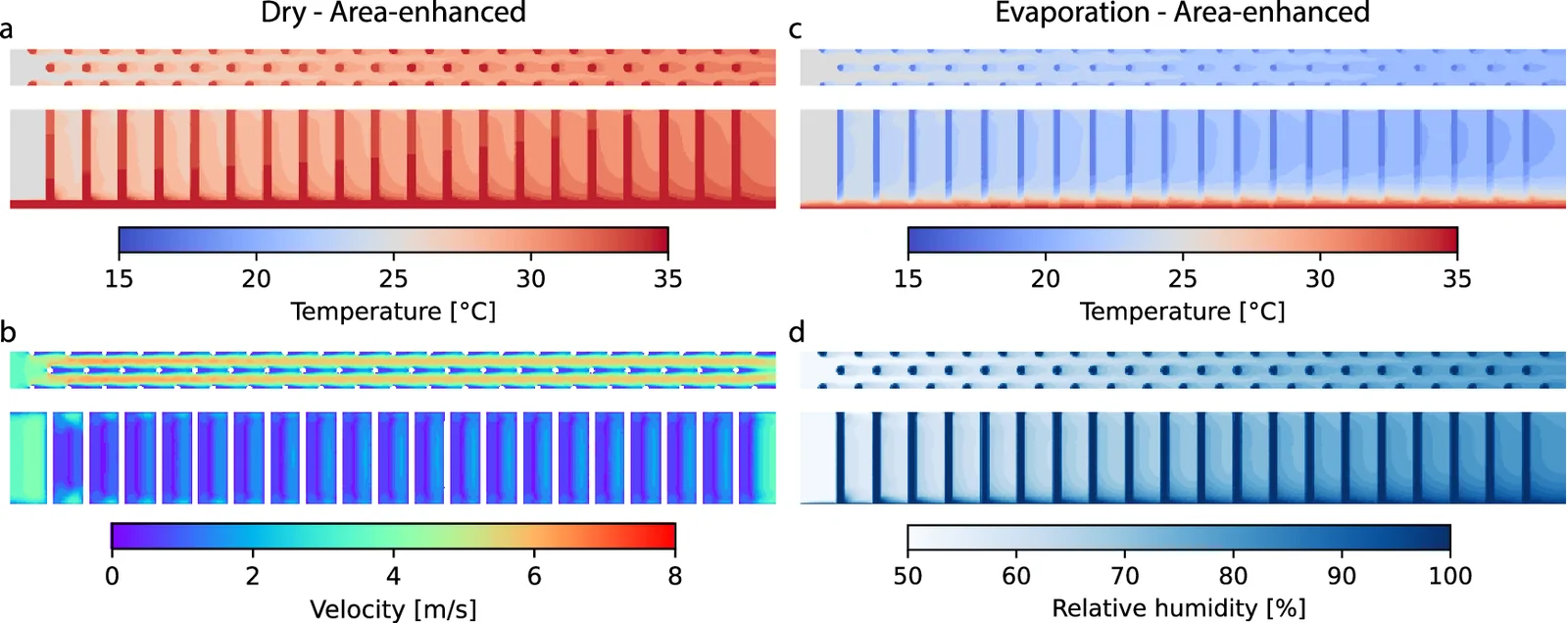
Pin-fin macrochannel, dry versus evaporative convection at a fan power density of 125 Wm^−2^. Top-view and side-view contour plot of the area-enhanced dry convection case: **a** temperature, **b** velocity magnitude, and of the area-enhanced evaporating hydrogel case: **c** temperature, **d** relative humidity. Contours are shown from a representative slice through the array: top-view plane parallel to the base at mid-gap height (*H*/2) and side-view streamwise plane at mid-width (*W*_*eff*_/2). Flow is left to right, and the base is assumed to be isothermal at 35 °C. The pin-fin array induces inter-fin acceleration, recirculation, and wakes that disrupt and locally thin down the thermal boundary layer, yielding large wall temperature gradients and high sensible heat extraction **a**, **b**. In **c**, **d**, the thin species boundary layer drives a high interfacial vapor flux and strong latent cooling; however, the hydrogel’s low thermal conductivity (*k*_hyd_ = 0.6 Wm^−1^ K^−1^) limits conduction from the interface to the base, so a significant fraction of the evaporative cooling appears as air-side cooling and humidification rather than increased base heat removal.

The effect of surface area enhancement is first assessed for the dry-convection case by adding pin fins to the macrochannel base. While added surface area and flow disturbance are expected to improve heat transfer at a given inlet velocity, pin fins also increase pressure drop. To account for this trade-off directly, we characterize performance through *q*_base_ versus *q*_fan_ so that the aerodynamic penalty associated with increased flow resistance is inherently included. As shown in Fig. 4, the dry area-enhanced configuration yields an approximately eightfold increase in heat transfer relative to the dry flat baseline at matched fan power (red curve versus orange curve), while the geometric area ratio is only about fourfold. This indicates that the improvement is driven not only by added surface area but also by boundary-layer disruption and enhanced mixing, which increase local convective heat and mass transfer coefficients. The associated temperature and velocity fields are shown in Fig. 5a, b, where inter-fin acceleration, recirculation regions, and wakes repeatedly thin the thermal boundary layer and sustain large wall temperature gradients. The same pin-fin geometry is then considered with a fully wetted hydrogel, representing a structured evaporative interface. In contrast to the dry case, Fig. 4 shows that the performance gain from area enhancement in the hydrogel evaporating configuration (dark green vs light green) is negligible and does not follow the classical behavior of extended surfaces. Indeed, despite the clear increase in evaporation rate inferred from the stronger air-side humidification and thinner local species boundary layers (Fig. 5c, d), the added area does not translate into a commensurate increase in *q*_base_.

This limited gain with area-enhanced hydrogel evaporators can be attributed to their internal thermal resistance. Because the hydrogel thermal conductivity is low (*k*_hyd_ ~ 0.6 Wm^−1^ K^−1^ in the model), the extended fins lengthen the conduction path between the isothermal base and the evaporating interface, reducing the effective utilization of added area. As a result, a significant fraction of the incremental latent heat extraction appears primarily as air-side cooling and humidification rather than increased heat removal at the base. This comparison identifies a first design rule: under evaporation, surface texturing improves panel-relevant performance only if the evaporating interface remains strongly thermally coupled to the base.

### Hydrogel overlayer on metal scaffold (HOMeS)

To overcome the hydrogel conduction bottleneck while retaining capillary-driven wetting and compatibility with thin panel form factors, we introduce the hydrogel overlayer on metal scaffold (HOMeS) concept. The HOMeS structure separates functions across pore scales and across thickness: a highly conductive and highly permeable porous metal scaffold (potentially made of sintered metal particles) provides efficient heat spreading from the base and low-hydraulic-resistance liquid distribution in the bulk, while a thin hydrogel layer at the external surface provides a continuous liquid-air interface and robust local rewetting. The bi-porous architecture and the surface hydrogel skin are illustrated in Fig. 1.

A key point is that, in this architecture, the hydrogel is not assumed to occupy the entire pore volume of the scaffold. Rather, the hydrogel is treated as a thin skin (and a near-surface interlocking fraction) whose primary role is capillary suction and interface wetting stabilization, while the internal pore space remains liquid-saturated during continuous operation. From the standpoint of effective thermal conductivity, both hydrogel and liquid water have similar thermal conductivities; therefore, whether the pore-filling phase is hydrogel or water does not materially change the predicted *k*_eff_ compared with the dominant influence of the metallic network. This is consistent with the modeling intent here: *k*_eff_ is used to represent heat spreading through a liquid-saturated porous metal backbone, while the hydrogel skin is treated separately through the interfacial evaporation boundary condition.

In the simulations, the porous metal scaffold is represented as a homogeneous, liquid-saturated effective conduction medium with thermal conductivity *k*_eff_ evaluated using the Maxwell–Eucken relation, assuming copper as the solid fraction intrinsic thermal conductivity, *k*_s_ = 400 Wm^−1^ K^−1^ (see *Methods* section). A representative porosity of *ε* = 40% is adopted, which is achievable with sintered copper scaffolds having pore sizes on the order of a few hundred micrometers.

**Fig. 6:**
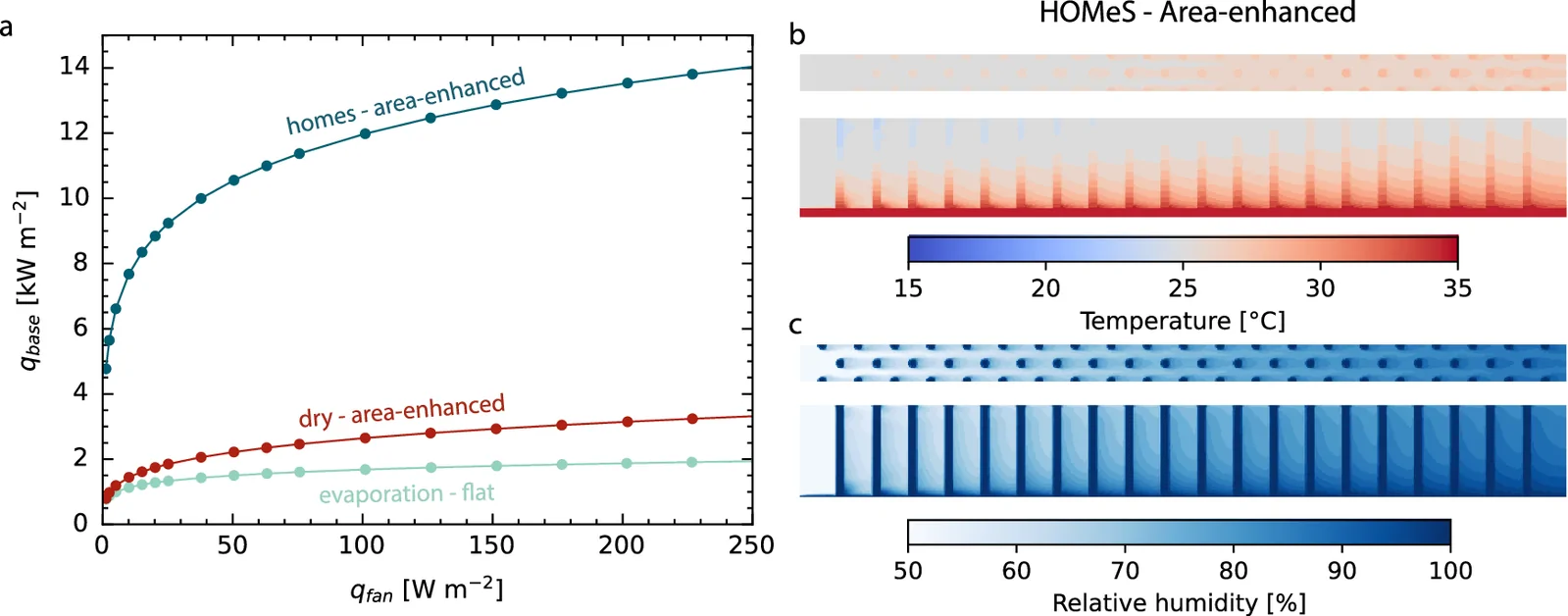
HOMeS area-enhanced macrochannel performance. **a** *q*_base_ versus *q*_fan_ for the HOMeS area-enhanced macrochannel, compared with the dry area-enhanced case and the flat evaporating macrochannel. At matched fan power, the HOMeS area-enhanced configuration delivers substantially higher performance (~4.5 × larger than the dry area-enhanced case and ~7 × larger than the flat evaporation case at a fan power density of 125 Wm^−2^). Contour maps of **b** air temperature and **c** relative humidity at a fan power density of 125 Wm^−2^ with a fully wetted area-enhanced HOMeS; flow is left to right. Fields are plotted in the same top-view and side-view through the pin-fin array. Due to the high effective thermal conductivity of the metal scaffold, the fins are now thermally coupled to the base, allowing area enhancement and inter-fin mixing to substantially improve the base heat transfer. Meanwhile, air humidification causes the relative humidity to approach saturation along the channel, limiting downstream evaporation.

Using this HOMeS effective medium in the same external pin-fin geometry and under the same baseline operation described in Fig. 2, and assuming a thin, fully wetted surface such that additional diffusive resistance across the gel is negligible compared with the convective resistance in the air, Fig. 6a reports *q*_base_ as a function of *q*_fan_. For reference, the flat evaporating macrochannel (*evaporation-flat*, in light green) and the area-enhanced dry macrochannel (*dry-area-enhanced*, in red) are included in Fig. 6a to highlight the benefits of the HOMeS concept compared to the dry area-enhanced case and the flat evaporating macrochannel. The sensitivity to the imposed base temperature *T*_base_ is reported in Supplementary Note S2. Because evaporative performance depends on the ambient state^31^, the sensitivity to the inlet air temperature *T*_in_ and relative humidity *ϕ*_in_ is mapped over the full operating envelope in Supplementary Note S3. In contrast to the hydrogel-only pin fins, the HOMeS textured configuration restores the expected benefit of surface area enhancement under evaporation: at *q*_fan_ = 125 Wm^−2^, *q*_base_ is approximately 4.5 × higher compared to the dry area-enhanced case and approximately 7 × higher compared to the flat evaporation case. This large gain indicates that, with thermal coupling between the evaporating surface and the heat source, added interfacial area and inter-fin mixing can indeed be translated into a higher dissipated base heat flux rather than only air-side cooling.

The corresponding temperature and relative humidity fields (Fig. 6b, c) show that the air approaches saturation rapidly along the channel for the baseline enhanced geometry at *q*_fan_ = 125 Wm^−2^. This saturation-limited behavior has two implications. First, it confirms that the enhanced interface is effectively utilized near the inlet where vapor driving forces remain high, consistent with the observed performance gains. Second, it suggests that additional geometric changes can provide further improvements by delaying the approach to saturation and/or reducing pressure drop, thereby increasing the amount of useful latent cooling obtained per unit fan power. Together with the hydrogel-only comparison (Fig. 4), these results sharpen the thermal-coupling design rule: surface texturing and flow mixing can improve evaporative macrochannel performance only when a highly conductive pathway connects the base to the evaporating interface, as provided here by a liquid-saturated porous metal scaffold.

Results above assume a continuous, fully wetted hydrogel skin with negligible residual thickness, such that any added resistance across the gel film is small compared with air-side transport and the thermal pathway remains dominated by the conductive scaffold. In practice, fabrication may instead leave local regions where the scaffold is exposed, reducing the effective wetted interfacial area, or a finite residual hydrogel overlayer above the scaffold introducing an additional resistance between the metal network and the interface. The performance sensitivity to the residual overlayer thickness is quantified in Supplementary Note S4.

### Effect of channel height on boundary-layer disruption

The preceding results indicate that, when thermal coupling to the evaporation interface is preserved, pin fins can deliver cooling gains that exceed the geometric area ratio, consistent with enhanced interfacial transport driven by boundary-layer disruption and flow mixing.

To test this mechanism, we retain the baseline pin array (*d*_pin_ = 2 mm, *s*_pin_ = 5 mm) and fix the pin height at *h*_pin_ = 25 mm, while increasing the macrochannel height from *H* = 25 to 450 mm. For *H* > *h*_pin_, a tip-clearance region forms above the array, reducing confinement, and progressively diminishing the interaction between the core flow and the textured interface as we keep *A*_interface_/*A*_footprint_ the same.

**Fig. 7:**
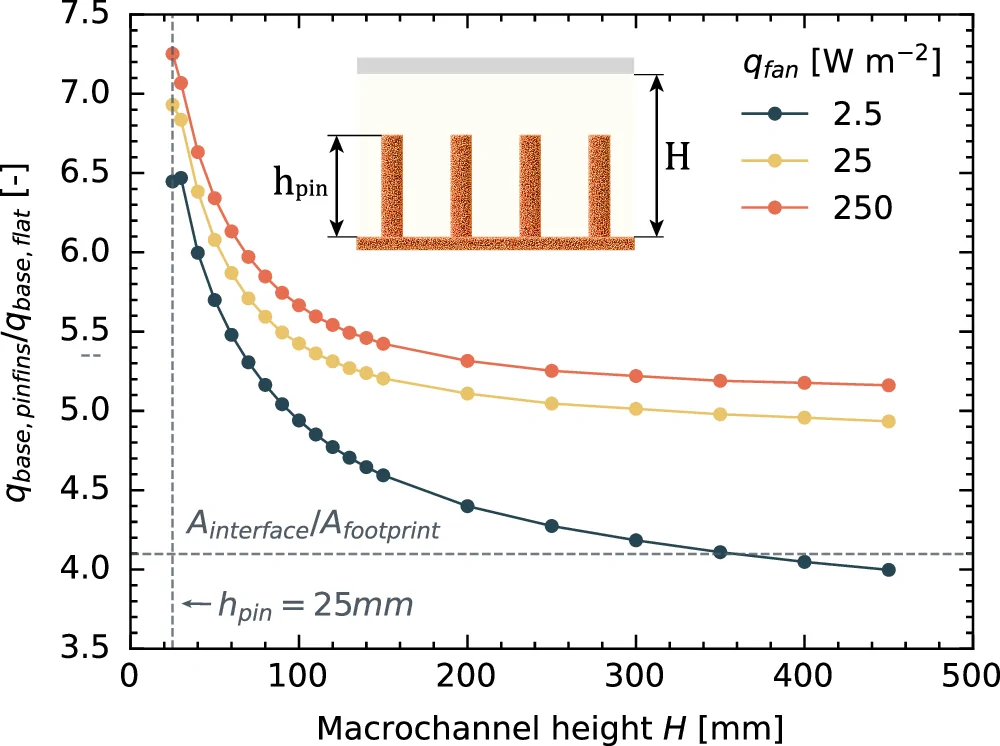
Effect of the macrochannel height on the base-cooling enhancement provided by pin fins. The enhancement factor *q*_base,pinfins_/*q*_base,flat_ is shown as a function of channel height *H* for a fixed pin array (*d*_pin_ = 2 mm, *s*_pin_ = 5 mm, *h*_pin_ = 25 mm) under fan-power constraints *q*_fan_ = 2.5, 25, and 250 Wm^−2^ (shown as dark teal, gold, and orange curves with filled circular markers, respectively), where *q*_base,flat_ denotes the corresponding flat evaporating macrochannel at the same *H*. The horizontal gray dashed line marks the interfacial area ratio *A*_interface_/*A*_footprint_ ≈ 4, and the vertical gray dashed line marks the baseline pin height *h*_pin_ = 25 mm. The inset shows the pin-fin geometry, with the pin height *h*_pin_ and macrochannel gap *H* indicated. Increasing *H* reduces the boundary-layer disruption, causing the enhancement to decay.

For each *H*, the inlet velocity is adjusted to satisfy a prescribed fan-power constraint, and the resulting base heat removal in the pin-fin channel is compared to that of a flat channel of identical footprint and height at matched fan power densities *q*_fan_ = 2.5, 25, and 250 Wm^−2^. The corresponding heat transfer enhancement factor, *q*_base,pinfins_/*q*_base,flat_, is reported in Fig. 7, which decreases with increasing *H* for all fan power levels. This is because boundary-layer disruption by pin fins becomes less significant as *H* increases. With small tip clearance, the boundary layer structure is predominantly determined by the channel geometry. With larger channel gaps compared to the pin height, a bypass region develops above the array, and the fraction of the flow interacting with the textured interface decreases. When *H* becomes sufficiently large, though, the inlet air velocity plays a more important role in governing the fluid boundary layer. Higher *q*_fan_ leads to higher inlet velocity and a thinner boundary layer. As a result, the decrease of *q*_base,pinfins_/*q*_base,flat_ is slower for *q*_fan_ = 250 Wm^−2^ and for larger *H*. In addition, it is worth noting that at *q*_fan_ = 2.5 Wm^−2^, the enhancement factor can fall to values even below *A*_interface_/*A*_footprint_ at the largest *H* considered, indicating that the added surface area is not fully utilized under the weak flow field and limited boundary layer disturbance. Overall, the results in Fig. 7 suggest that the zero-clearance design (*h*_pin_ = *H*) should be adopted when optimizing the pin-fin geometries for the panel-level heat transfer performance.

### Pin-fin geometry design under a fan-power constraint

Having established that a thermally conductive HOMeS translates surface area enhancement into increased *q*_base_ under evaporation, we examine how the external pin-fin geometry controls performance for a fixed panel footprint and thickness. The macrochannel footprint and gap height are fixed to the baseline values (*W* = 200 mm, *L* = 200 mm, *H* = 25 mm; see Fig. 2), and the objective is to identify pin arrays that maximize the dissipated base heat flux *q*_base_ at a specified fan power density *q*_fan_ (*h*_pin_ is set to *H* = 25 mm based on the results obtained in Fig. 7). This design problem is strongly coupled because the same geometric changes may simultaneously vary the interfacial area, boundary-layer mixing, and channel pressure drop (i.e., flow rate at fixed fan power) as well as the streamwise development of air temperature and humidity (which sets the evaporation driving force) and fin conduction length scales that govern thermal utilization of the added area. We consider *d*_pin_ and *s*_pin_ independently over the range 1–15 mm and 1–20 mm respectively in 1 mm increments (a 15 × 20 grid), yielding 300 candidate geometries. We restrict the number of pin pitches that can be packed within the *W* × *L* footprint to be an integer.

**Fig. 8:**
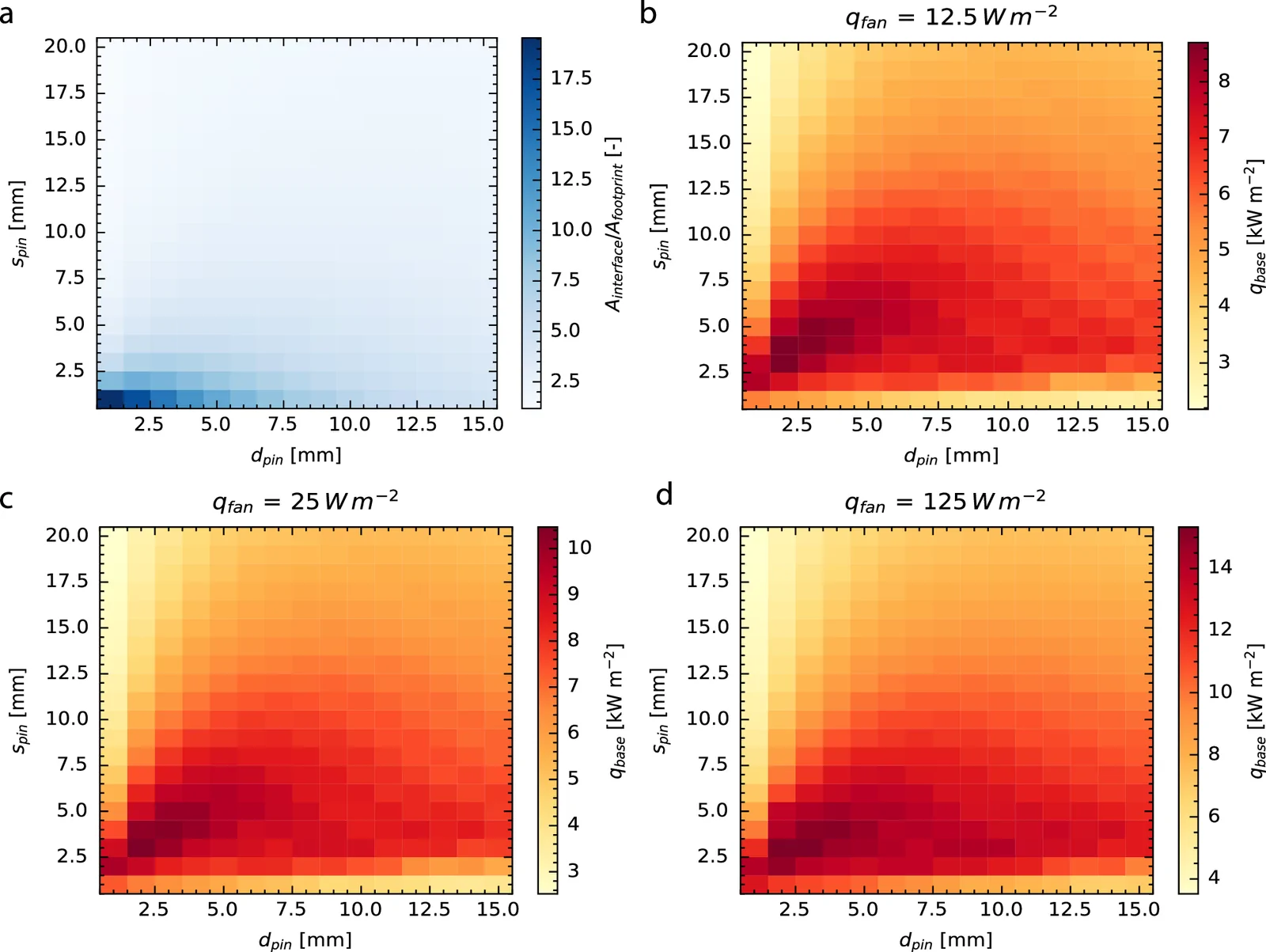
Fan-power-constrained geometry maps in (*d*_pin_, *s*_pin_) space for a HOMeS area-enhanced macrochannel with fixed pin height *h*_pin_ = *H.* **a** Interfacial area ratio *A*_interface_/*A*_footprint_ as a function of pin diameter *d*_pin_ and edge-to-edge spacing *s*_pin_ (pitch *p* = *d*_pin_ + *s*_pin_). Dissipated base heat flux *q*_base_ evaluated at three fixed fan power densities: **b** *q*_fan_ = 12.5 Wm^−2^, **c** *q*_fan_ = 25 Wm^−2^, and **d** *q*_fan_ = 125 Wm^−2^. Maps are displayed as discrete cells corresponding to the 1 mm sampling grid and integer pin packing within a fixed footprint. Decreasing pitch increases interfacial area but also increases flow resistance, yielding a non-monotonic dependence of *q*_base_ on geometric area ratio.

Figure 8 a reports the corresponding map of interfacial area ratio, *A*_interface_/*A*_footprint_, over the (*d*_pin_, *s*_pin_) design space. As expected, the area ratio increases strongly as the pitch *p* = *d*_pin_ + *s*_pin_ decreases, reflecting the increased pin number density and the resulting rise in total wetted sidewall area per footprint. Over the parameter range considered, the largest area ratios therefore occur for the smallest pitches (simultaneously small *d*_pin_ and small *s*_pin_). Along cuts of fixed *s*_pin_ or fixed *d*_pin_, the area ratio does not vary perfectly smoothly because changing *d*_pin_ and *s*_pin_ simultaneously modifies (i) the lateral area per pin, (ii) the pin number density, and (iii) the exposed base area between pins, in addition to the discrete pin-count constraint discussed above. This purely geometric context is useful, but it is not sufficient to predict performance because the fan-power constraint penalizes high-flow-resistance layouts and the effective utilization of the added area depends on fin conduction and on the streamwise decay of the evaporation driving force.

Figure 8b–d presents contour maps of the dissipated base heat flux *q*_base_ over the (*d*_pin_, *s*_pin_) design space for fan power densities *q*_fan_ = 12.5, 25, and 125 Wm^−2^. Because *s*_pin_ is defined as the edge-to-edge spacing, decreasing either *d*_pin_ or *s*_pin_ decreases the pitch *p* = *d*_pin_ + *s*_pin_, increases the number density of pins that can be packed in a fixed footprint, and generally increases the wetted interfacial area per footprint (Fig. 8a). The maps show that higher *q*_base_ is indeed obtained in a broad region of relatively small pitches, i.e., denser pin arrays. However, *q*_base_ does not increase monotonically as the arrays are made denser. At fixed *q*_fan_, very small spacings (tight arrays) are penalized because the associated increase in flow resistance shifts the operating point to a lower volumetric flow rate, reducing the overall heat and mass transfer rates even though the geometric area is larger. At the opposite extreme, large pitches (large *d*_pin_ and/or large *s*_pin_) lead to sparse arrays with lower interfacial area density and correspondingly lower *q*_base_. As a result, each fan-power level exhibits a broad optimum region in the (*d*_pin_, *s*_pin_) space rather than a monotonic trend with area ratio. As *q*_fan_ increases from 12.5 to 125 Wm^−2^, the high-performance region extends toward smaller *s*_pin_ (and thus smaller pitch), consistent with the ability of higher available fan power to sustain adequate flow through the channel even with denser arrays. Across the fan-power range shown, the maxima remain located in the small-to-moderate pitch regime (here, *d*_pin_ ~ 2–3 mm and *s*_pin_ ~ 3–4 mm in Fig. 8). In the next section, these dense sweep maps serve as a baseline for benchmarking Bayesian optimization (BO), enabling a direct assessment of how rapidly BO can recover near-optimal designs in the same (*d*_pin_, *s*_pin_) space.

### Bayesian optimization for constrained geometry search

Grid-based parametric sweeps provide an initial view of the fan-power-constrained design maps, but the computational cost increases rapidly as additional geometric degrees of freedom are introduced. Bayesian optimization (BO) was therefore employed as an active-learning alternative for identifying high-performing HOMeS macrochannel geometries using a limited number of CFD evaluations. The BO studies first considered the same HOMeS area-enhanced macrochannel configuration, boundary conditions, and fan-power coupling as in the preceding two-parameter design maps (Fig. 8), such that the only change was the sampling strategy used to explore the design space. The design variables remain the pin diameter *d*_pin_ and edge-to-edge spacing *s*_pin_, while the pin height was fixed at *h*_pin_ = *H* (no tip clearance) and the fan power density was held fixed at *q*_fan_ = 125 Wm^−2^.

**Fig. 9:**
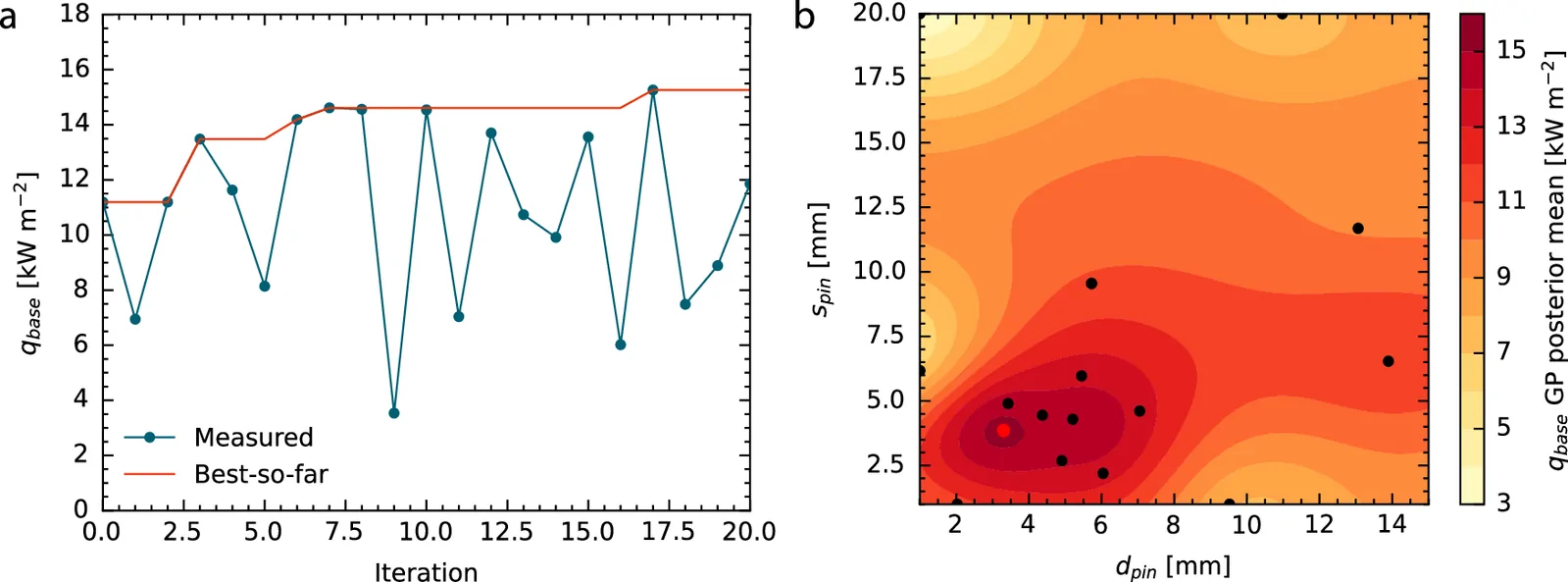
Bayesian optimization in the two-parameter (*d*_pin_, *s*_pin_) design space for the HOMeS area-enhanced macrochannel with fixed pin height *h*_pin_ = *H* (no tip clearance), identical to the preceding design-map configuration. **a** Convergence history of the dissipated base heat flux *q*_base_ (measured CFD evaluations) and the best-so-far value versus iteration. **b** GP surrogate posterior mean of *q*_base_ over the design space at the final iteration. Black markers denote evaluated designs, and the red marker indicates the best observed design.

The optimization objective was maximizing the dissipated base heat flux, *q*_base_, evaluated at the fixed fan power density *q*_fan_. Figure 9a shows the measured objective values and the corresponding best-so-far sequence. Despite the non-monotonicity inherent to exploration, the best design improves rapidly and then stabilizes, indicating that BO localizes the high-performance region (see Fig. 8d) with only 20 CFD evaluations, compared with the full 15 × 20 grid (300 geometries) used to generate the grid map. The corresponding Gaussian-process (GP) surrogate is visualized in Fig. 9b through its posterior mean over the (*d*_pin_, *s*_pin_) plane. The inferred optimum region is consistent with the grid-sweep trend, concentrating toward relatively small *d*_pin_ and *s*_pin_ under the present fan-power constraint.

**Fig. 10:**
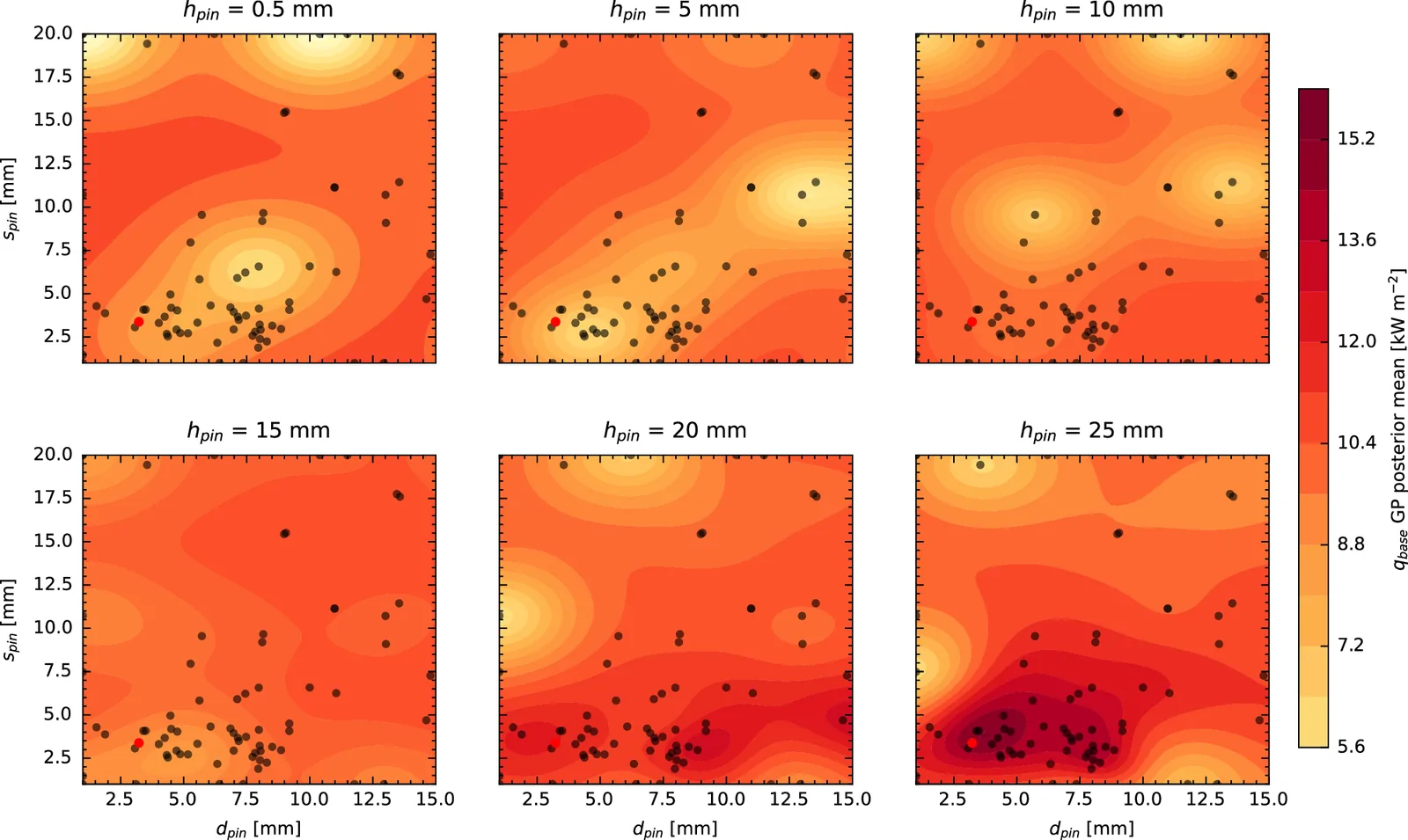
Three-parameter Bayesian optimization including pin height *h*_pin_ (tip-clearance control), shown as GP surrogate slices after 90 iterations. Contours represent the GP posterior mean of *q*_base_ in the (*d*_pin_, *s*_pin_) plane for fixed values of *h*_pin_; *h*_pin_ = *H* = 25 mm corresponds to zero tip clearance. Black markers indicate evaluated designs, and the red marker denotes the best observed design. The highest predicted performance is concentrated at small *d*_pin_ and *s*_pin_ and is most pronounced for *h*_pin_ near *H*, consistent with the two-parameter optimum region.

The BO framework was next extended to include tip clearance by treating the pin height *h*_pin_ as an additional design variable, with the clearance defined by *H* − *h*_pin_. This three-parameter formulation retains the same objective, maximizing *q*_base_ at fixed *q*_fan_ and the same thermofluid configuration as above. In this case, we allow the aerodynamic and transport consequences of partial-height pins to be explored directly. Because the surrogate landscape is now three-dimensional, the GP posterior mean is visualized by two-dimensional slices in the (*d*_pin_, *s*_pin_) plane at fixed *h*_pin_ values (Fig. 10). After 90 BO iterations, the high-performance region remains concentrated at small *d*_pin_ and *s*_pin_, and the strongest predicted performance occurs as *h*_pin_ approaches *H*, consistent with the no-tip-clearance guideline suggested by Fig. 7. Overall, this BO framework provides a direct route to optimize in higher-dimensional design spaces (which may consider manufacturability and architectural integrability) without exhaustive CFD sweeps.

## Discussion

In summary, we developed a fan-power-constrained framework to evaluate facade-scale evaporative macrochannel panels and used it to design the HOMeS architecture that reconciles evaporation enhancement, pressure-drop penalties, and internal conduction limits. Using *q*_base_ versus *q*_fan_ as the primary metric, flat evaporation already provides a strong latent-heat benefit, yielding ≈ 5 × higher *q*_base_ than a dry flat macrochannel at *q*_fan_ = 125 Wm^−2^. For dry convection, pin fins increase *q*_base_ by ≈ 8 × relative to the dry flat baseline at matched *q*_fan_, exceeding the geometric area ratio *A*_interface_/*A*_footprint_ ≈ 4 because the pin-fin array disturbs the near-wall transport layers. In contrast, under evaporation, hydrogel pin fins provide little improvement in *q*_base_ despite stronger air-side cooling. This breakdown of extended-surface behavior is consistent with thermal under-utilization of fins: the low thermal conductivity of hydrogels (*k*_hyd_ = 0.6 Wm^−1^ K^−1^) limits the internal thermal conduction between the isothermal base and the evaporating interface.

The HOMeS architecture restores thermal coupling by separating functions across pore scales: a liquid-saturated porous metal scaffold spreads heat and distributes liquid with low loss, while a thin hydrogel skin stabilizes a continuous wetted interface. As a result, the textured HOMeS recovers extended-surface gains under evaporation and reaches ≈ 7 × higher *q*_base_ than a flat evaporating channel at *q*_fan_ = 125 Wm^−2^. This area-enhancement benefit persists across the fan-power range, including at low fan power density (Supplementary Note S6). Design maps show a non-monotonic dependence of *q*_base_ on *A*_interface_, with the optimal design near *d*_pin_ ~ 2–3 mm and *s*_pin_ ~ 3–4 mm for our baseline macrochannel footprint of *W* = 200 mm and *L* = 200 mm, reflecting the trade-off between interfacial area and channel pressure drop. We have shown that Bayesian optimization can identify similar optima with substantially fewer CFD evaluations, and suggests zero tip clearance to maximize boundary layer disturbance, consistent with the results from dedicated parametric studies.

Although the present model is primarily intended for geometric design ranking under consistent assumptions, it is instructive to contextualize the potentially achievable cooling performance. For the baseline 200 × 200 mm panel operated at *q*_fan_ = 125 Wm^−2^, the best BO design yields a base heat flux *q*_base_ ≈ 15 kWm^−2^. At this operating point, the parasitic fan-power density of 125 Wm^−2^ is below 1% of the rejected heat, corresponding to an effectiveness *q*_base_/*q*_fan_ ≈ 120. This illustrates the high-flux potential of thermally coupled evaporative texturing when latent heat extraction is not limited by internal conduction. However, it cannot be extrapolated to other panel sizes by simple area scaling: under a fan-power constraint, pressure drop and flow rate vary nonlinearly with geometry, and performance must be evaluated at the target footprint and parasitic-power budget. For this reason, and to facilitate comparison with the literature, we report the base heat removal and fan power as areal power densities normalized by the panel footprint. This areal normalization is rigorous only for tiling identical optimized panels (each with its own fan and fresh inlet air), not for enlarging a single panel, because under a fan-power constraint *q*_base_ and *q*_fan_ do not scale linearly with footprint.

On a building facade, absorbed solar irradiance adds a parasitic heat load that depends on facade orientation and on the reflectivity of the panel’s outward-facing surface. With a solar-reflective coating (reflectance 0.7, readily achievable with standard cool-facade materials^32^), even the worst-case orientation (west-facing, peak irradiance ~750 Wm^−2^) yields an absorbed load of only ~225 Wm^−2^, about 1.5% of the optimized HOMeS cooling capacity (~15 kWm^−2^) reported here, a secondary effect at the design operating point.

The hydrogel skin is adopted because it offers a scalable route to a fully wetted, high-area evaporating surface: its intrinsic micro- to nanoporous network sustains strong capillary suction, and it can be deposited over large areas by simple conformal coating, in contrast to the more demanding fabrication of an all-metal nanoporous skin^33,34^.

Generally, the results establish a clear design rule: evaporative surface texturing improves panel-level cooling only when a highly conductive pathway connects the base to the evaporation interface. When this coupling is weak, added interfacial area primarily increases air-side humidification rather than useful base heat removal. Because the objective is defined directly in terms of *q*_base_ versus *q*_fan_, the same Bayesian optimization workflow can be applied at any prescribed panel size to identify geometries that balance interfacial area, pressure drop, and boundary-layer disruption. This fan-power-constrained methodology provides a physically consistent route for designing high-flux, facade-integrated evaporative panels.

## Methods

### Numerical model and computational domain

All conjugate heat-mass transfer simulations were performed using the finite-volume solver FLUENT (ANSYS Inc.). Geometry generation, meshing, and solver setup (materials, boundary conditions, and post-processing quantities) were automated using Python interfaces to ANSYS tools and executed on a high-performance computing cluster. Steady-state solutions were obtained for all cases reported in the main text. To verify the solver independently of the discretization approach, the finite-volume FLUENT predictions were cross-checked against an independent finite-element implementation in COMSOL Multiphysics for the baseline area-enhanced HOMeS configuration, with close agreement in the base heat removal and fan power (see Supplementary Fig. S10).

The full macrochannel panel consists of a periodic array of pin-fin unit cells in the spanwise direction. To reduce computational cost while retaining the key three-dimensional transport mechanisms (inter-fin acceleration, wake formation, and mixing), the computational domain represents a single longitudinal row of the full channel and exploits symmetry of the rotated square pin lattice (Fig. S8). For a pin diameter *d*_pin_ and spacing *s*_pin_, the effective width of one longitudinal row is

1 $${W}_{{\rm{eff}}}=2\sin \left(\frac{\pi }{4}\right)\left({s}_{{\rm{pin}}}+{d}_{{\rm{pin}}}\right).$$

The corresponding width ratio

2 $${f}_{w}=\frac{W}{{W}_{{\rm{eff}}}},$$

relates the real macrochannel width *W* to the simulated row width and is used to scale heat removal and fan power to the full panel footprint.

In the streamwise direction, the domain contains an integer number of pin unit cells. The simulated effective length is

3 $${L}_{{\rm{eff}}}={N}_{{\rm{cell}}}{W}_{{\rm{eff}}}+{d}_{{\rm{pin}}},$$

where the additional *d*_pin_ avoids a sharp entrance/exit edge at the active-section boundaries and

4 $${N}_{{\rm{cell}}}=\left\lfloor \frac{L}{{W}_{{\rm{eff}}}}\right\rfloor .$$

The length ratio

5 $${f}_{l}=\frac{L}{{L}_{{\rm{eff}}}},$$

accounts for the small mismatch between the real length *L* and the realizable periodic length *L*_eff_. Unless otherwise stated, all pin fins span the full macrochannel height (*h*_pin_ = *H*), i.e., there is no tip clearance.

Flat macrochannel configurations were simulated in 2D in the streamwise/wall-normal plane. Area-enhanced configurations were simulated in 3D to resolve flow development around pin fins and the associated heat and mass transfer enhancement.

### Governing equations for moist-air flow and species transport

The macrochannel flow was modeled as a steady, weakly compressible mixture of dry air and water vapor. The mixture density *ρ*_m_, specific heat *c*_p,m_, thermal conductivity *k*_m_, and viscosity *μ*_m_ were treated as an ideal gas mixture with temperature-dependent intrinsic properties of dry air and water vapor^35–37^. The mixture density was calculated using the ideal gas law. Buoyancy forces were neglected because the operating regime is dominated by forced convection.

Mixture mass conservation was enforced as

6 $$\nabla \cdot ({\rho }_{m}{\bf{v}})=0,$$

where *ρ*_*m*_ is the mixture density and **v** is the mean velocity vector. The steady Reynolds-averaged momentum equation was solved as

7 $${\rho }_{m}({\bf{v}}\cdot \nabla ){\bf{v}}=-\nabla p+\nabla \cdot \bar{\tau },$$

with *p* the static pressure and $\bar{\tau }$ the Reynolds-averaged stress tensor,

8 $$\bar{\tau }={\mu }_{{\rm{eff}}}\left(\nabla {\bf{v}}+{(\nabla {\bf{v}})}^{T}\right)-\frac{2}{3}{\mu }_{{\rm{eff}}}(\nabla \cdot {\bf{v}}){\bf{I}},\qquad {\mu }_{{\rm{eff}}}={\mu }_{m}+{\mu }_{t},$$

where *μ*_*m*_ is the mixture viscosity and *μ*_*t*_ is the turbulent viscosity predicted by the turbulence model.

The steady energy equation for the moist-air mixture was written as

9 $${\rho }_{m}{c}_{p,m}{\bf{v}}\cdot \nabla T+\nabla \cdot {\bf{q}}=0,$$

where *c*_*p*,*m*_ is the mixture specific heat and **q** is the thermal diffusion flux,

10 $${\bf{q}}=-{k}_{{\rm{m,eff}}}\nabla T,\qquad {k}_{{\rm{m,eff}}}={k}_{m}+{k}_{t},$$

with *k*_*m*_ the mixture laminar thermal conductivity and *k*_*t*_ the turbulent thermal conductivity.

Transport of water vapor was modeled using a convection–diffusion equation for the vapor mass fraction *Y*_*v*_,

11 $${\rho }_{m}{\bf{v}}\cdot \nabla {Y}_{v}+\nabla \cdot {\bf{j}}=0,$$

with diffusive flux

12 $${\bf{j}}=-{\rho }_{m}{D}_{{\rm{eff}}}\nabla {Y}_{v},\qquad {D}_{{\rm{eff}}}={D}_{va}+{D}_{t},$$

where *D*_*va*_ is the binary diffusion coefficient of water vapor in air and *D*_*t*_ is the turbulent diffusion coefficient obtained from the turbulence model.

The flow spans laminar to turbulent regimes depending on fan power and geometry. To provide a consistent closure across this range and to resolve near-wall gradients of temperature and vapor mass fraction, all cases were computed using the *k*–*ω* SST model, which provides *μ*_*t*_ and the associated turbulent transport coefficients^38^.

### Heat conduction and effective material models

In all non-fluid regions (base plate and pin structures), only steady heat conduction was solved:

13 $${\nabla }^{2}T=0,$$

with constant thermal conductivity assigned within each material region.

Hydrogel was modeled as a homogeneous medium with thermal conductivity set equal to that of liquid water, *k*_hyd_ = 0.6 Wm^−1^ K^−1^. Liquid transport resistance within the hydrogel was neglected in the CFD model; interfacial evaporation was therefore governed by convective and diffusive transport in the air phase, and the hydrogel was treated as a continuously wetted medium in the idealized limit where internal supply resistance is small^39^.

For HOMeS cases, the pin region was represented as a liquid-saturated porous copper scaffold and treated as a homogeneous effective conduction medium. The effective thermal conductivity assigned to the scaffold region was estimated using the Maxwell–Eucken effective-medium model^40^,

14 $${k}_{{\rm{eff}}}={k}_{{\rm{s}}}\frac{2{k}_{{\rm{s}}}+{k}_{{\rm{f}}}-2\varepsilon ({k}_{{\rm{s}}}-{k}_{{\rm{f}}})}{2{k}_{{\rm{s}}}+{k}_{{\rm{f}}}+\varepsilon ({k}_{{\rm{s}}}-{k}_{{\rm{f}}})}\approx {k}_{{\rm{s}}}\frac{2(1-\varepsilon )}{2+\varepsilon }\qquad {\rm{for}}\,{k}_{{\rm{s}}}\,\gg\, {k}_{{\rm{f}}},$$

where *k*_s_ is the intrinsic thermal conductivity of the solid fraction taken as pure copper (*k*_s_ = 400 Wm^−1^ K^−1^), *k*_f_ is the conductivity of the pore-filling phase (taken as liquid water; *k*_f_ ≈ *k*_hyd_), and *ε* is porosity. The corresponding *k*_eff_(*ε*) trend is reported in Fig. S7 and indicates that the scaffold remains strongly thermally conductive relative to hydrogel, preserving thermal coupling between the base and the evaporating interface. Because water and the hydrogel were assigned comparable thermal conductivities, the pore-filling phase had a negligible influence on the effective conductivity relative to the metallic network. Residual hydrogel overlayer effects were treated separately by explicitly resolving a concentric hydrogel annulus of thickness *t*_layer_ around the conductive scaffold core (see Supplementary Note S4).

### Boundary conditions and interfacial evaporation model

To allow the flow to develop before entering the active heated/evaporative section and to improve numerical stability, entrance and exit extensions of length *L*_in_ = 100 mm and *L*_out_ = 100 mm were appended upstream and downstream of the active region (Fig. S9). At the inlet, the moist-air mixture entered at a prescribed temperature *T*_in_ and relative humidity *ϕ*_in_ at 1 atm, with a uniform velocity profile of magnitude *U*_in_. The outlet was set to zero gauge pressure, *p*_out_ = 0 Pa. The pressure drop used for fan-power evaluation, Δ*p*_section_, was measured across the active macrochannel section only, excluding the entrance and exit extensions. The base of the structure was held isothermal at *T*_base_. The opposite wall (and the pin-fin tips in the area-enhanced cases) was treated as no-slip, adiabatic, and impermeable: **v** = 0, − **q** ⋅ **n** = 0, − **j** ⋅ **n** = 0. At the liquid-air interface (flat hydrogel surface or HOMeS surface), the wall was assumed fully wetted and no-slip. The vapor mass fraction at the interface was fixed to its saturation value at the local interface temperature,

15 $${Y}_{v,{\rm{int}}}={Y}_{{\rm{sat}}}({T}_{{\rm{int}}}),$$

where *Y*_sat_ was computed from the saturation vapor pressure *p*_sat_(*T*_int_) and the ideal-gas mixture relation at total pressure *p*. The interface is treated as a macroscopic fully wetted surface held at saturation, and the sub-pore menisci within the hydrogel are not resolved. Because the evaporative flux is limited by air-side vapor diffusion and these pores are far smaller than the air-side boundary layers, this micro-scale interfacial area is not expected to change the predictions, and neglecting it is in any case conservative because it adds no additional evaporating area to the model. Evaporation couples species transport to energy transport at the interface through the latent heat flux. The interfacial energy balance enforces conservation of heat,

16 $${q}_{{\rm{evap}}}+{q}_{{\rm{conv}}}+{q}_{{\rm{cond}}}=0,$$

where *q*_cond_ is the conductive heat flux from the solid/effective medium to the interface, *q*_conv_ is the sensible heat flux between the interface and the air, and *q*_evap_ is the latent heat flux associated with the evaporative vapor mass flux. Using Fourier’s and Fick’s laws, the fluxes are written as

17 $${q}_{{\rm{conv}}}=-{k}_{{\rm{m,eff}}}\nabla T\cdot {\bf{n}},\qquad {q}_{{\rm{cond}}}=-{k}_{s}\nabla T\cdot {\bf{n}},$$

18 $${q}_{{\rm{evap}}}=-{h}_{lv}({T}_{{\rm{int}}}){\rho }_{m}{D}_{{\rm{eff}}}\nabla {Y}_{v}\cdot {\bf{n}},$$

where *h*_*lv*_(*T*_int_) is the latent heat of vaporization at the interface temperature and **n** is the unit normal pointing into the air domain.

The governing equations were discretized using the finite-volume method and solved using the pressure-based steady solver in FLUENT. Gradients were reconstructed using a least-squares cell-based method, and convective terms in the momentum, energy, turbulence, and vapor species equations were discretized using second-order upwind schemes. A pseudo-time-stepping approach was employed to improve robustness of the tightly coupled heat–mass transfer at a saturated interface. A grid sensitivity analysis is reported in the Supplementary Note S5.

### Fan power coupling and performance metrics

To compare different surface geometries under a realistic parasitic-power constraint, the inlet velocity *U*_in_ was not prescribed directly. Instead, for each configuration and target fan power density, *U*_in_ was adjusted iteratively until the fan power density computed from the simulated pressure drop matched the specified value.

The fan power density associated with a given solution, normalized by the panel footprint area, is evaluated as

19 $${q}_{{\rm{fan}}}=\frac{1}{{\eta }_{{\rm{fan}}}{A}_{{\rm{footprint}}}}\left[{f}_{l}{{\Delta }}{p}_{{\rm{section}}}+\frac{1}{2}({k}_{{\rm{in}}}+{k}_{{\rm{out}}}){\bar{\rho }}_{m}{U}_{{\rm{in}}}^{2}\right]{f}_{w}{A}_{{\rm{cs,in}}}{U}_{{\rm{in}}},$$

where *A*_footprint_ = *WL* is the panel footprint area, Δ*p*_section_ is the simulated pressure drop across the active section, scaled by the length ratio *f*_*l*_, *k*_in_ and *k*_out_ are inlet/outlet loss coefficients, ${\bar{\rho }}_{m}$ is the active-section volume-averaged mixture density, *A*_cs,in_ is the inlet cross-sectional area, and *η*_fan_ is an overall fan efficiency. A flat plenum entrance and sudden discharge were assumed, with *k*_in_ = 0.5 and *k*_out_ = 1.0^41^. The fan efficiency was set to *η*_fan_ = 0.3 and treated as constant over the operating range.

The dissipated base heat flux follows from the conductive heat flux through the isothermal base of the simulated row, scaled to the full panel by the symmetry factors *f*_*w*_ and *f*_*l*_ and normalized by the footprint area *A*_footprint_:

20 $${q}_{{\rm{base}}}=\frac{{f}_{w}{f}_{l}}{{A}_{{\rm{footprint}}}}\int_{{A}_{{\rm{base}}}}\left(-{k}_{s}\nabla T\cdot {\bf{n}}\right)dA,$$

where *A*_base_ is the base area in the simulated row.

### Bayesian optimization (BO) of geometry under a fan-power constraint

Geometry optimization is posed as a bounded maximization problem:

21 $${\mathop{\max }\limits_{{\bf{x}}\in {{\Omega }}}}\,J({\bf{x}})\equiv {q}_{{\rm{base}}}({\bf{x}};{q}_{{\rm{fan}}}),$$

where **x** collects the geometric design variables (e.g., *d*_pin_, *s*_pin_, *h*_pin_), Ω denotes bound constraints (feasible ranges), and the fan power density *q*_fan_ is held fixed. BO builds a probabilistic surrogate of the expensive CFD objective from the set of completed evaluations ${{\mathcal{D}}}_{n}={\{({{\bf{x}}}_{i},{J}_{i})\}}_{i = 1}^{n}$. We use a Gaussian-process (GP) regression surrogate, which provides at each iteration *n* a posterior predictive distribution for *J*(**x**) with mean *μ*_*n*_(**x**) and standard deviation *σ*_*n*_(**x**)^42^. The next design is selected by maximizing an acquisition function that balances exploitation (high *μ*_*n*_) and exploration (high *σ*_*n*_). We use the Expected Improvement (EI) acquisition with exploration parameter *ξ* = 0.01,

22 $${{\rm{EI}}}_{n}({\bf{x}})={\mathbb{E}}\left[\max \left(0,\,J({\bf{x}})-{J}^{* }_{n}-\xi \right)\right],$$

where ${J}_{n}^{* }={\mathop{\max }\nolimits_{i\,\le\, n}}\;{J}_{i}$ is the best observed objective value. Because the GP posterior predictive distribution at any fixed **x** is Gaussian, EI admits the closed form

23 $${{\rm{EI}}}_{n}({\bf{x}})=\left({\mu }_{n}({\bf{x}})-{J}_{n}^{* }-\xi \right){{\Phi }}(Z)+{\sigma }_{n}({\bf{x}})\phi (Z),\qquad Z=\frac{{\mu }_{n}({\bf{x}})-{J}_{n}^{* }-\xi }{{\sigma }_{n}({\bf{x}})},$$

with Φ( ⋅ ) and *ϕ*( ⋅ ) the standard normal cumulative distribution function (CDF) and probability density function (PDF) (and EI_*n*_ = 0 when *σ*_*n*_ = 0). The next query point is then

24 $${{\bf{x}}}_{n+1}=\arg {\mathop{\max }\limits_{{\bf{x}}\in {{\Omega }}}}\,{{\rm{EI}}}_{n}({\bf{x}}).$$

In practice, an initial set of designs is sampled within Ω to initialize the surrogate, and subsequent points are proposed by EI maximization while avoiding exact duplicates (within a specified rounding tolerance). For each BO query **x**_*n*_, a CFD case is executed (with inlet conditions adjusted so that the specified *q*_fan_ is met), and the panel-level objective is extracted as the base heat flux, that is, the base cooling power scaled to the full footprint by the symmetry factors *f*_*w*_ and *f*_*l*_ and normalized by the footprint area *A*_footprint_:

25 $$J({{\bf{x}}}_{n})={q}_{{\rm{base}}}({{\bf{x}}}_{n})=\frac{{q}_{{\rm{base,row}}}({{\bf{x}}}_{n})\,{f}_{w}{f}_{l}}{{A}_{{\rm{footprint}}}}.$$

This definition ensures consistency between the optimization objective and the performance metric reported in the results.

The BO figures are generated from the sequence of evaluated designs {(**x**_*i*_, *J*_*i*_)} and the corresponding GP surrogate used for visualization. The convergence plot reports (i) the measured objective *J*_*i*_ at each iteration and (ii) the best-so-far sequence ${J}_{i}^{* }={\mathop{\max }\nolimits_{k\,\le\, i}}\;{J}_{k}$ versus iteration index. To visualize the surrogate-predicted objective landscape, the GP posterior mean is evaluated on a uniform 60 × 60 grid over the parameter bounds and plotted as a two-dimensional contour map in the design space, with evaluated designs overlaid (and the best-so-far design highlighted in red). When three design variables are present, the surrogate visualization is reported as a stack of two-dimensional slices at selected fixed values of the third variable (see Fig. 10).

## Supplementary information

Supplementary Information

## Data Availability

The datasets generated and/or analyzed during the current study are not publicly available due to the proprietary nature of the in-house simulation pipeline used to generate them, but are available from the corresponding author on reasonable request.

## Code availability

The code generated and/or analyzed during the current study is not publicly available due to the proprietary nature of the in-house simulation pipeline, but is available from the corresponding author on reasonable request.
