# Supplementary material for: Design and modeling of area-enhanced, bi-porous, evaporative macrochannel for building facade cooling
Source: Npj Therm Sci Eng. 2026 Oct 7;1(1):19. doi: 10.1038/s44435-026-00021-6 (PMC13671269; doi:10.1038/s44435-026-00021-6)
Supplement: Supplementary file 1 [file 44435_2026_21_MOESM1_ESM.pdf]

Supplementary Information

Design and modeling of area-enhanced, bi-porous, evaporative macrochannel for building facade cooling

Gautier Rouaze[1], Zhengmao Lu[1,*]

[1] EPFL, Lausanne, Switzerland

*Corresponding author: zhengmao.lu@epfl.ch

Note S1. Wetting design rule based on liquid-supply pressure losses

The CFD model assumes a fully wetted evaporative surface, implying that capillary-fed liquid transport through the hydrogel/metal composite can sustain the evaporative mass flux predicted by the flow simulations without dryout. To assess this assumption and establish a thickness guideline for the hydrogel overlayer, a reduced-order liquid pressure-drop model is formulated for the same operating condition considered above. The composite is idealized as a porous metal scaffold capped by a hydrogel layer composed of an interlocked region of thickness δ_{int} and an external overlayer of thickness δ_{over} (Fig. S1a). Because the liquid is supplied at the base and must wick up through the metal scaffold and pins to reach the evaporating skin, the relevant scaffold flow length is set by the pin height, $L_{\text{scaf}} \approx h_{\text{pin}} \approx 25$ mm, rather than the base-plate thickness. The local evaporation demand is set as $j_{\text{evap}} = 4\ \text{g}\,\text{m}^{-2}\,\text{s}^{-1}$ (representative value extracted from the CFD results), which requires a superficial liquid supply velocity $u_\ell = j_{\text{evap}}/\rho_\ell$, where ρ_ℓ is the liquid density.

Representative numerical inputs used to generate the illustrative pressure-drop curves in Fig. S1b are summarized in Table S1. These values are order-of-magnitude estimates and are intended to highlight the trend and the existence of an optimum overlayer thickness, rather than to provide a precise prediction for a specific material batch.

Quantity	Symbol	Representative value
Evaporation demand	j_{evap}	$4\ \text{g}\,\text{m}^{-2}\,\text{s}^{-1}$
Hydrogel permeability	K_{gel}	$1.0 \times 10^{-17}\ \text{m}^2$
Scaffold flow length	L_{scaf}	25 mm
Interlocked thickness	δ_{int}	200 μm
Overlayer thickness range	δ_{over}	5–500 μm
Scaffold porosity	$\varepsilon_{\text{scaf}}$	0.4
Particle diameter	$d_{p,\text{scaf}}$	200 μm

Table S1: Representative parameter values used in the reduced-order wetting pressure-drop model. Values are approximate and are used to illustrate the existence of an optimum δ_{over} rather than to provide an exact prediction.

The total liquid-side pressure drop is decomposed as

$$\Delta p_{\text{tot}} = \Delta p_{\text{scaf}} + \Delta p_{\text{int}} + \Delta p_{\text{over},\perp} + \Delta p_{\text{over},\parallel}, \tag{S1}$$

where Δp_{scaf} is the pressure drop through the metal scaffold, Δp_{int} is the pressure drop through the hydrogel interlocked region, $\Delta p_{\text{over},\perp}$ is the cross-plane pressure drop through the hydrogel overlayer, and $\Delta p_{\text{over},\parallel}$ is the in-plane pressure drop through the hydrogel overlayer.

A one-dimensional Darcy model gives

$$\Delta p_{\text{scaf}} = \mu_\ell \frac{j_{\text{evap}}}{\rho_\ell} \frac{L_{\text{scaf}}}{K_{\text{scaf}}}. \tag{S2}$$

where μ_ℓ is the liquid dynamic viscosity, and K_{scaf}, the scaffold permeability estimated using the Kozeny–Carman relation for sintered particles [1]

$$K_{\text{scaf}} = \frac{\varepsilon_{\text{scaf}}^3 d_{p,\text{scaf}}^2}{C_k \left(1 - \varepsilon_{\text{scaf}}\right)^2}, \tag{S3}$$

with $\varepsilon_{\text{scaf}}$, the scaffold porosity, $d_{p,\text{scaf}}$, the characteristic particle diameter, and $C_k = 180$. For the parameter range considered here, $K_{\text{scaf}} \gg K_{\text{gel}}$, so even with the full pin-height travel distance $L_{\text{scaf}} \approx 25$ mm the associated scaffold pressure drop remains at least an order of magnitude smaller than the hydrogel losses. Consequently, the liquid supply is not expected to constrain the use of area-enhanced metal geometries (e.g., pin-fin scaffolds); the dominant flow resistance arises within the hydrogel layer. The interlocked region is treated as a parallel hydrogel/void composite with an effective permeability $K_{\text{eff,int}} = \varepsilon_{\text{scaf}} K_{\text{gel}}$, giving

$$\Delta p_{\text{int}} = \mu_\ell \frac{j_{\text{evap}}}{\rho_\ell} \frac{\delta_{\text{int}}}{K_{\text{eff,int}}}. \tag{S4}$$

The cross-plane loss through the overlayer is

$$\Delta p_{\text{over},\perp} = \mu_\ell \frac{j_{\text{evap}}}{\rho_\ell} \frac{\delta_{\text{over}}}{K_{\text{gel}}}. \tag{S5}$$

In addition to cross-plane transport, the overlayer must redistribute liquid laterally to replenish evaporation sites. Axisymmetric radial Darcy flow is assumed within a film of thickness δ_{over} with a uniform sink term $j_{\text{evap}}/(\rho_\ell \delta_{\text{over}})$, yielding

$$\frac{1}{r}\frac{d}{dr}\left(r\frac{dp}{dr}\right) = \frac{\mu_\ell j_{\text{evap}}}{K_{\text{gel}} \rho_\ell \delta_{\text{over}}}. \tag{S6}$$

With $dp/dr = 0$ at $r = 0$ and $p(R) = 0$ at a characteristic replenishment radius $R = d_{p,\text{scaf}}/2$, the associated in-plane pressure drop becomes

$$\Delta p_{\text{over},\parallel} = \frac{\mu_\ell j_{\text{evap}} d_{p,\text{scaf}}^2}{16 K_{\text{gel}} \rho_\ell \delta_{\text{over}}}. \tag{S7}$$

This term scales as $\Delta p_{\text{over},\parallel} \propto \delta_{\text{over}}^{-1}$, whereas $\Delta p_{\text{over},\perp} \propto \delta_{\text{over}}$. Their competition produces an optimum overlayer thickness that minimises Δp_{tot} (Fig. S1b): overly thin overlayers incur a large spreading resistance, whereas overly thick overlayers increase the cross-plane Darcy resistance.

The maximum sustainable liquid pressure head in a fully wetted hydrogel is bounded by the Young–Laplace pressure at the interface,

$$\Delta p_{\text{cap}} = \frac{2\sigma_{\ell v} \cos\theta_r}{r_{\text{pore}}}, \tag{S8}$$

where $\sigma_{\ell v}$ is the liquid–vapour surface tension, r_{pore} is a characteristic pore radius, and θ_r is the receding contact angle. For $\theta_r \approx 0^\circ$ and typical hydrogel pore sizes r_{pore} on the order of tens of nanometers, Δp_{cap} is in the MPa range and is therefore expected to exceed the viscous pressure losses through the hydrogel for the conditions investigated here.

Note S2. Effect of the base temperature

The imposed base temperature T_{base} is the isothermal temperature of the panel base plate and represents the coupling to an external liquid-side loop. A value near 35 °C corresponds to the condenser side of a vapor-compression cycle, whereas lower values correspond to the

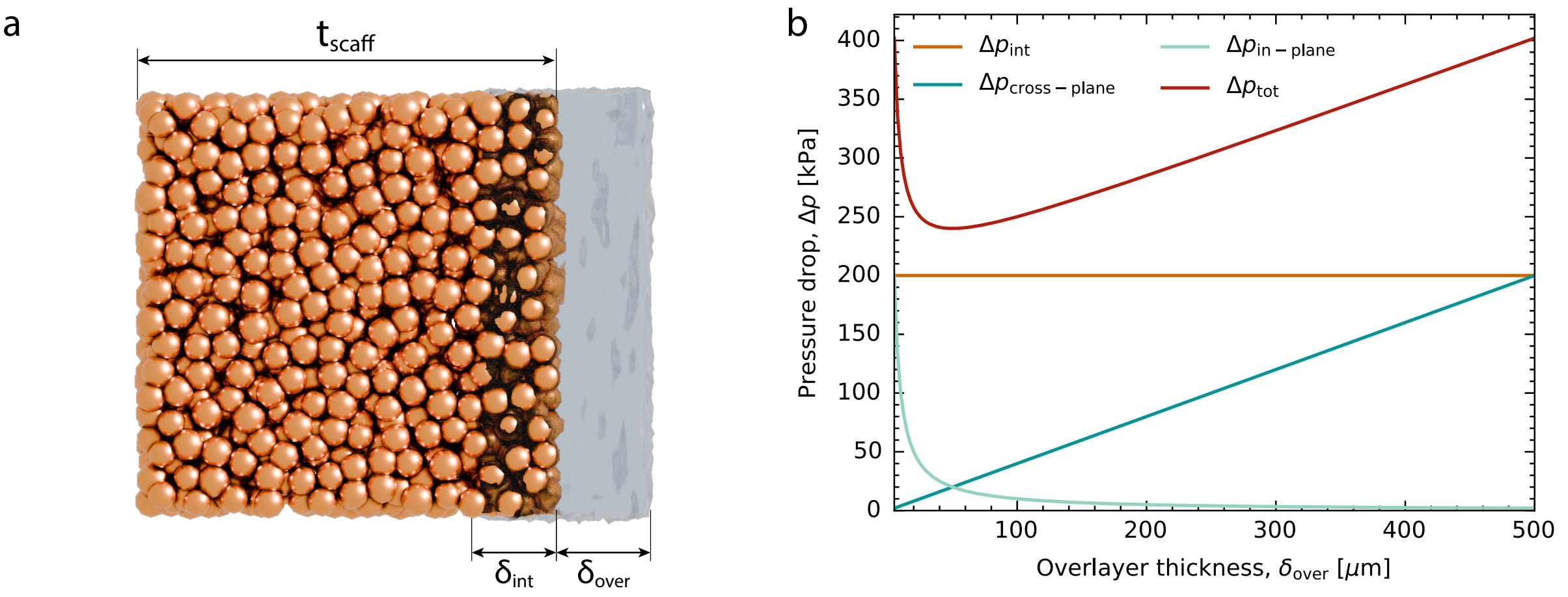

Fig. S1: Liquid-supply pressure losses and optimal hydrogel overlayer thickness. (a) Rendering of the idealized bi-porous composite: a liquid-saturated porous copper scaffold (thickness t_{scaff}) capped by an interlocked hydrogel region (thickness δ_{int}) and an external hydrogel overlayer (thickness δ_{over}). (b) Liquid-supply pressure losses as a function of hydrogel overlayer thickness δ_{over} for a representative evaporation demand $j_{\mathrm{evap}} = 4\ \mathrm{g\,m^{-2}\,s^{-1}}$ extracted from the CFD results. The cross-plane Darcy loss through the overlayer ($\Delta p_{\mathrm{over},\perp}$, teal curve) increases with δ_{over}, whereas the in-plane spreading loss ($\Delta p_{\mathrm{over},\|}$, light-green curve) decreases with δ_{over}, yielding a minimum in the total pressure drop (Δp_{tot}, red curve) at an intermediate thickness. The interlocked hydrogel region contributes a thickness-independent cross-plane loss (Δp_{int}, orange curve; δ_{int} fixed). The scaffold contribution is negligible relative to the hydrogel losses and is omitted for clarity.

direct supply of a cooled liquid stream for space or process cooling. The range $T_{\mathrm{base}} = 25$–$50\ ^\circ\mathrm{C}$ explored here spans these cases and reflects the design trade-off set by the cooling load and the downstream system. For the baseline HOMeS pin-fin macrochannel and the default inlet condition (Fig. 2), T_{base} was varied from 25 to 50 °C to generate an operating map of the base heat flux q_{base} under fan-power constraints $q_{\mathrm{fan}} = 2.5$, 25, and 250 $\mathrm{Wm^{-2}}$. Fig. S2 shows that q_{base} increases monotonically with T_{base} for all fan-power levels. Increasing T_{base} strengthens the sensible driving force ($T_{\mathrm{base}} - T_{\mathrm{in}}$) and increases the interfacial saturation vapor concentration, thereby enhancing both the sensible and latent heat transfer. At fixed T_{base}, higher q_{fan} yields larger q_{base} because increased flow rates raise transport coefficients and delay streamwise warming and humidification. The relevant T_{base} range is application-dependent, spanning the warm-coolant and condenser return temperatures encountered when the panel is used to produce cooled liquid for downstream heat exchangers (e.g. vapor-compression-cycle condensers).

Note S3. Effect of the inlet air properties

The sensitivity of the evaporative cooling panel performance to the inlet air properties, namely the relative humidity ϕ_{in} and dry-bulb temperature T_{in}, was assessed for the baseline HOMeS pin-fin macrochannel geometry (see Fig. 2). The inlet relative humidity was varied first, at fixed inlet temperature. Simulations were performed over $0.05 \leq \phi_{\mathrm{in}} \leq 0.95$ while maintaining the same dry-bulb temperature and base temperature as in the main study. The inlet velocity was adjusted to satisfy the prescribed fan-power constraints ($q_{\mathrm{fan}} = 2.5$, 25, and 250 $\mathrm{Wm^{-2}}$). Fig. S3a shows that the base heat flux q_{base} decreases approximately linearly over the range considered and for all selected q_{fan}. This trend reflects the reduction of the vapor-transport driving force between the saturated interface and the bulk flow as ϕ_{in} increases, which suppresses

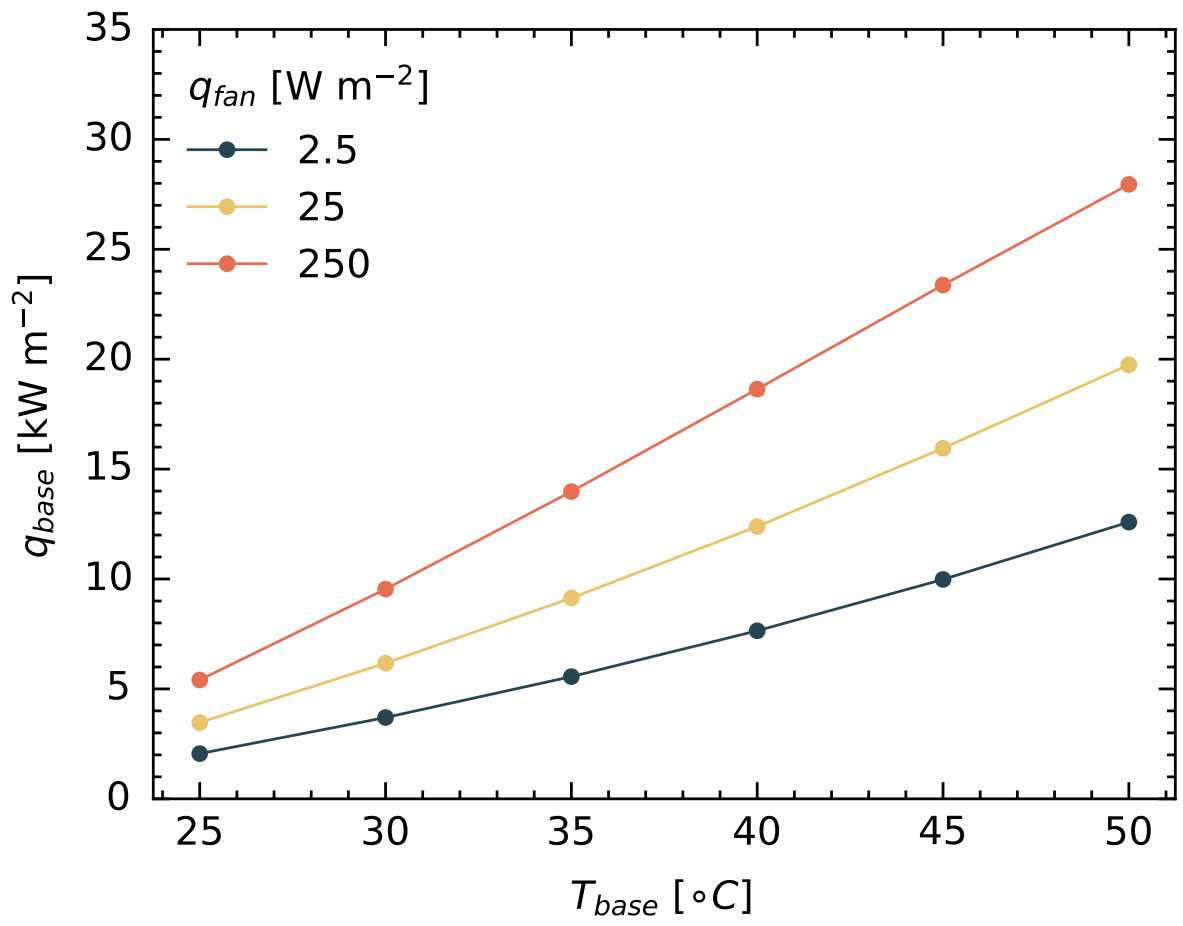

Fig. S2: Effect of imposed base temperature on the performance of the baseline HOMeS pin-fin macrochannel under the default inlet condition (Fig. 2). The dissipated base heat flux q_{base} is plotted as a function of the base temperature T_{base} over 25–50 °C, for three fan-power densities $q_{\text{fan}} = 2.5$, 25, and 250 Wm^{-2} (shown as dark teal, gold, and orange curves with filled circular markers, respectively).

evaporation and the associated latent heat extraction. In addition, increasing ϕ_{in} causes the flow to reach saturation earlier in the channel, so a smaller portion of the wetted surface remains evaporation-active, an effect that is most pronounced at low q_{fan} where the residence time is longest. Consequently, the quantitative dependence of q_{base} on ϕ_{in} is geometry-specific and is expected to vary with the wetted-surface area and streamwise length.

The dependence on inlet temperature was assessed separately by varying T_{in} from 10 to 40 °C at fixed $\phi_{\text{in}} = 0.5$, under the same fan-power constraints. As shown in Fig. S3b, q_{base} decreases monotonically with T_{in} for every fan level, because a warmer inlet both reduces the sensible driving force $T_{\text{base}} - T_{\text{in}}$ and, at fixed relative humidity, raises the inlet vapor loading and thereby lowers the evaporative (latent) driving force. Notably, the panel continues to provide net cooling even when the inlet is warmer than the base plate: at $T_{\text{in}} = 40$ °C, which is 5 °C above $T_{\text{base}} = 35$ °C, q_{base} remains positive for all three fan powers, equal to 1.9, 3.1, and 4.7 kWm^{-2} at $q_{\text{fan}} = 2.5$, 25, and 250 Wm^{-2} respectively. In this regime the sensible flux has reversed and warms the base, but at $\phi_{\text{in}} = 0.5$ the evaporative latent cooling more than compensates, so the panel still removes heat overall.

To characterise the combined effect of humidity and temperature, q_{base} was mapped over the $(\phi_{\text{in}}, T_{\text{in}})$ plane at the baseline $q_{\text{fan}} = 125$ Wm^{-2} (Fig. S3c). The base heat flux is largest for cold, dry inlets ($q_{\text{base}} \approx 19$ kWm^{-2} at $T_{\text{in}} = 10$ °C, $\phi_{\text{in}} = 0.05$) and decreases smoothly toward warm, humid inlets, consistent with the one-dimensional trends in panels (a) and (b). Net cooling is maintained across the entire mapped envelope, up to the base temperature itself: at $T_{\text{in}} = 35$ °C $= T_{\text{base}}$, q_{base} ranges from about 13.7 kWm^{-2} at $\phi_{\text{in}} = 0.05$ down to about 0.7 kWm^{-2} at $\phi_{\text{in}} = 0.95$, where the warm and humid corner approaches the cooling cutoff. Physically, raising either ϕ_{in} or T_{in} erodes the interface-to-bulk vapor-concentration difference that drives evaporation, but provided the inlet stays sufficiently dry the latent cooling remains large enough to keep the net base flux positive even up to and beyond T_{base}, as shown for $\phi_{\text{in}} = 0.5$ in panel (b).

Finally, the enhancement factor of the area-enhanced HOMeS channel relative to the flat evaporating channel, $q_{\text{base,AE}}/q_{\text{base,flat}}$, is mapped over the same plane in Fig. S3d, restricted to $T_{\text{in}} \leq 35$ °C where the flat reference still delivers net cooling and the ratio is well defined. The ratio is nearly uniform, remaining between about 6.8 and 7.0 across the entire humidity

and temperature range, a variation of only a few percent. This near-invariance arises because $\phi_{\rm in}$ and $T_{\rm in}$ set the inlet thermodynamic state, and hence the local driving forces, but they do not alter the area-enhancement and boundary-layer-mixing mechanism that distinguishes the HOMeS channel from the flat one. The absolute fluxes therefore shift with the inlet conditions while their ratio does not. This confirms that the $\sim 7\times$ benefit reported in the main text is a robust property of the architecture across the operating envelope, rather than an artefact of the nominal $T_{\rm in} = 25$ °C, $\phi_{\rm in} = 0.5$ condition.

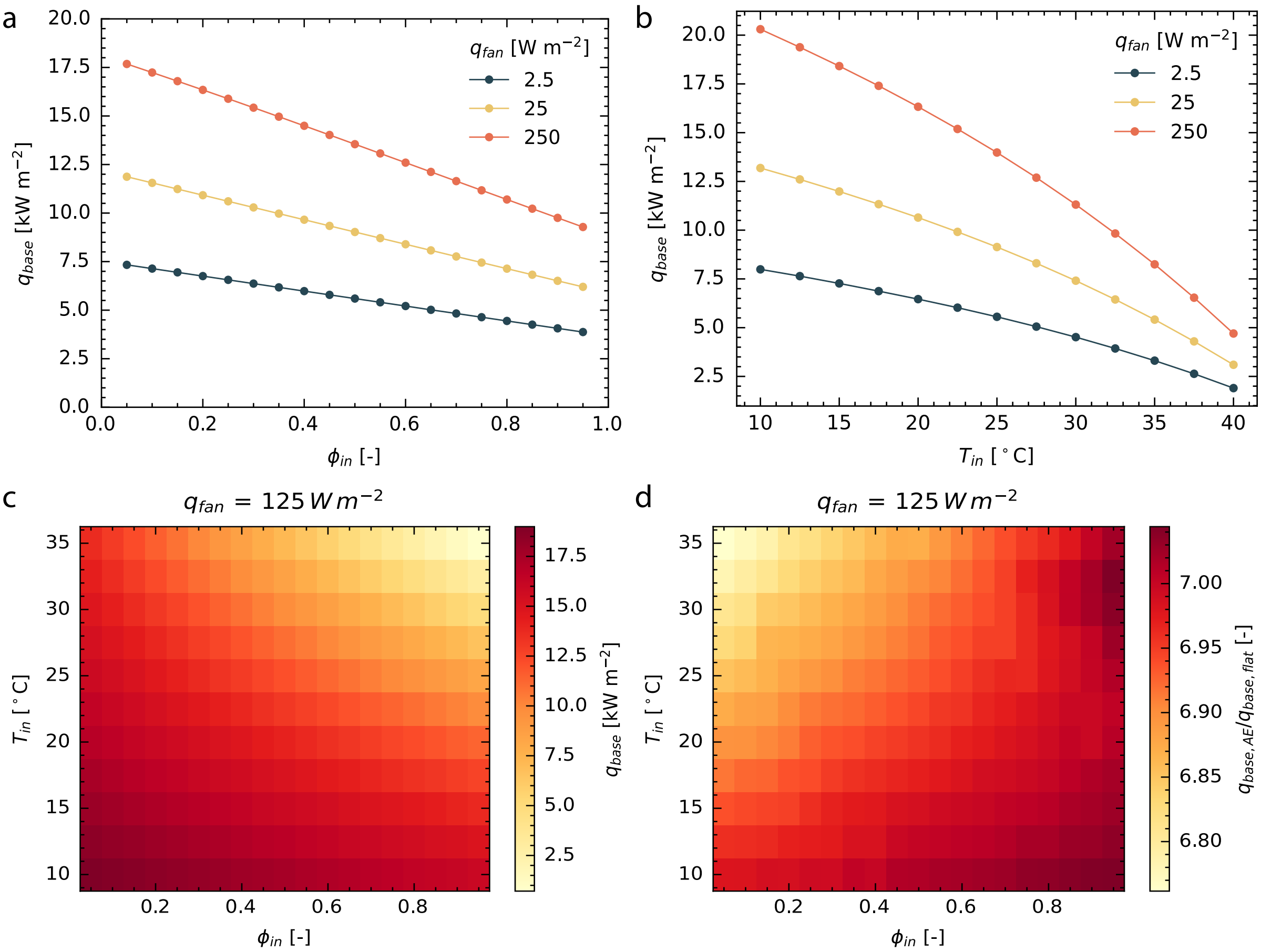

Fig. S3: Effect of the inlet air properties on the evaporative macrochannel performance for the baseline HOMeS pin-fin geometry. (a) $q_{\rm base}$ as a function of inlet relative humidity $\phi_{\rm in}$ for fan-power constraints $q_{\rm fan} = 2.5$, 25, and 250 $\rm Wm^{-2}$ (dark teal, gold, and orange curves, respectively). (b) $q_{\rm base}$ as a function of inlet temperature $T_{\rm in}$ at fixed $\phi_{\rm in} = 0.5$ for the same three fan-power levels and colours. Net cooling persists above the base temperature $T_{\rm base} = 35$ °C, with $q_{\rm base} > 0$ at $T_{\rm in} = 40$ °C. (c) Map of $q_{\rm base}$ over the $(\phi_{\rm in}, T_{\rm in})$ plane at $q_{\rm fan} = 125$ $\rm Wm^{-2}$. (d) Map of the enhancement factor $q_{\rm base,AE}/q_{\rm base,flat}$ over the same plane (shown for $T_{\rm in} \leq 35$ °C, where the flat reference still delivers net cooling), which stays close to $7\times$ (between about 6.8 and 7.0), demonstrating that the HOMeS enhancement is insensitive to the inlet air conditions.

Note S4. Effect of surface hydrogel overlayer thickness

The simulations of pin-fin HOMeS channels discussed assume an infinitesimally thin, continuous, fully wetted hydrogel skin at the scaffold surface, such that any additional resistance across the gel film is negligible compared with the air-side transport resistance and the thermal pathway remains dominated by the conductive metal scaffold. In practice, fabrication can deviate from

this idealization; manufacturable infusion processes may leave a finite residual overlayer above the scaffold that introduces additional thermal resistance between the metal network and the evaporating interface. Importantly, the residual overlayer thickness (above the external scaffold surface) is distinct from the interlocked hydrogel that penetrates into the porous scaffold to provide mechanical anchoring and hydraulic continuity.

To quantify the impact of a residual overlayer, the external pin geometry (d_{pin} and s_{pin}) is held fixed so that the velocity field remains comparable across cases. A concentric composite pin is then considered, in which a hydrogel annulus of thickness t_{layer} progressively replaces a porous-metal core of diameter $d_{\mathrm{pin}} - 2t_{\mathrm{layer}}$ (baseline $d_{\mathrm{pin}} = 2$ mm), as sketched in Fig. S4a. This idealized construction captures the combined effect of (i) increasing the low-k conduction length from the scaffold to the interface and (ii) reducing the cross-sectional fraction of the high-conductivity scaffold, while keeping the external blockage constant. The thickness t_{layer} is varied from 0 mm (vanishing overlayer, corresponding to the ideal HOMeS limit, see Fig. 6) to 1.0 mm (a full hydrogel pin, see Fig. 4) in increments of 0.2 mm.

Figure S4b reports q_{base} versus q_{fan} for the different t_{layer} values. Cooling performance decreases monotonically as t_{layer} increases, because the low-conductivity hydrogel lengthens the thermal path between the conductive scaffold/base and the evaporating interface, partially negating the benefit of the high-k pathway. Even modest residual thicknesses produce noticeable degradation, emphasizing that HOMeS performance depends on preserving a short thermal path from the scaffold to the evaporation surface. These results establish a thermal design constraint for HOMeS fabrication: any residual hydrogel overlayer above the scaffold should be kept as thin as practicable to preserve thermal coupling between the conductive network and the evaporating interface.

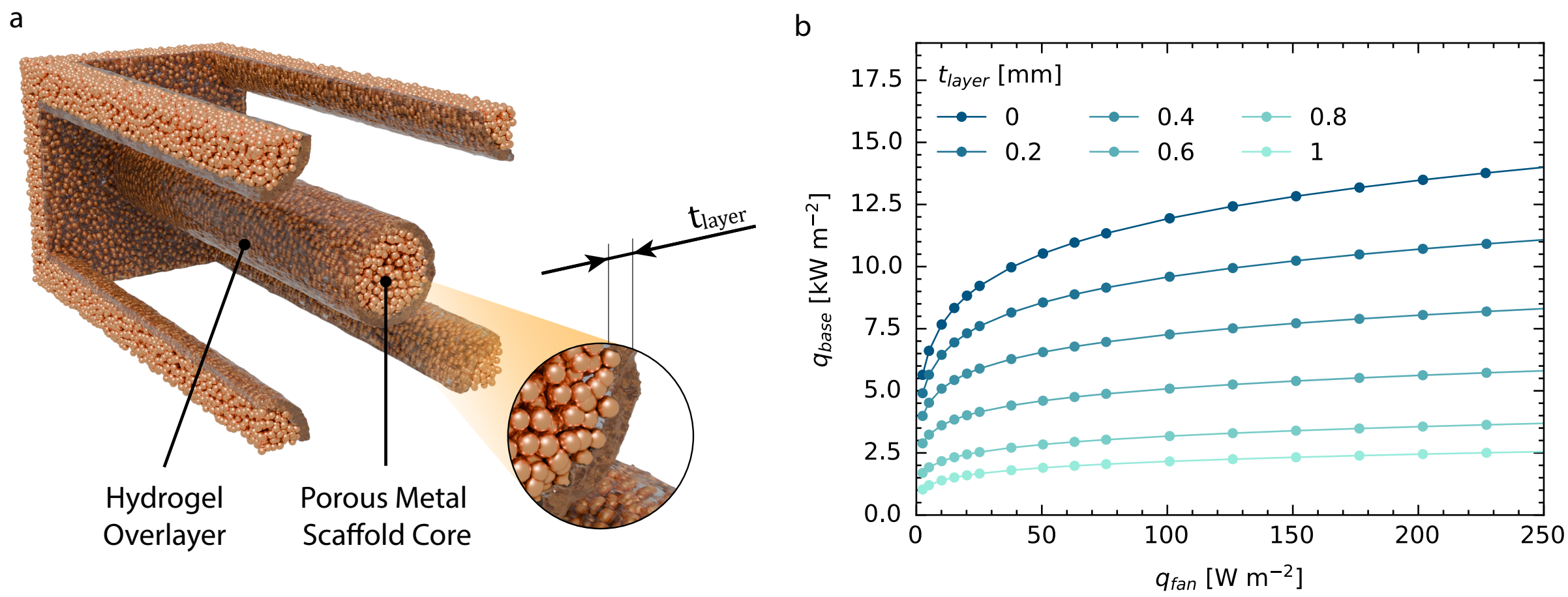

Fig. S4: Effect of residual hydrogel overlayer thickness on HOMeS performance. (a) Schematic of a composite pin in which a porous-metal core is coated by a residual hydrogel overlayer of thickness t_{layer}. (b) Dissipated base heat flux q_{base} versus fan power density q_{fan} for $t_{\mathrm{layer}} =$ 0, 0.2, 0.4, 0.6, 0.8, 1.0 mm, shown as successive coloured curves of increasing overlayer thickness as labelled in the legend. Cooling performance decreases monotonically with increasing t_{layer} because the low-conductivity hydrogel increases the internal conduction resistance between the evaporating interface and the isothermal base, partially negating the conductive benefit of the scaffold. These results highlight the need to minimize the residual overlayer thickness to preserve the high-k pathway.

Note S5. Mesh dependence analysis

A grid-sensitivity analysis was carried out for the HOMeS configuration with the baseline pin-fin geometry (see Fig. 2) by systematically refining the mesh and monitoring the resulting changes in q_{base} and q_{fan}. Figure S5 indicates that a mesh with approximately 1.7×10^6 control volumes is sufficient for the case considered, as further refinement produces only minor variations in the predicted base heat flux and fan power density.

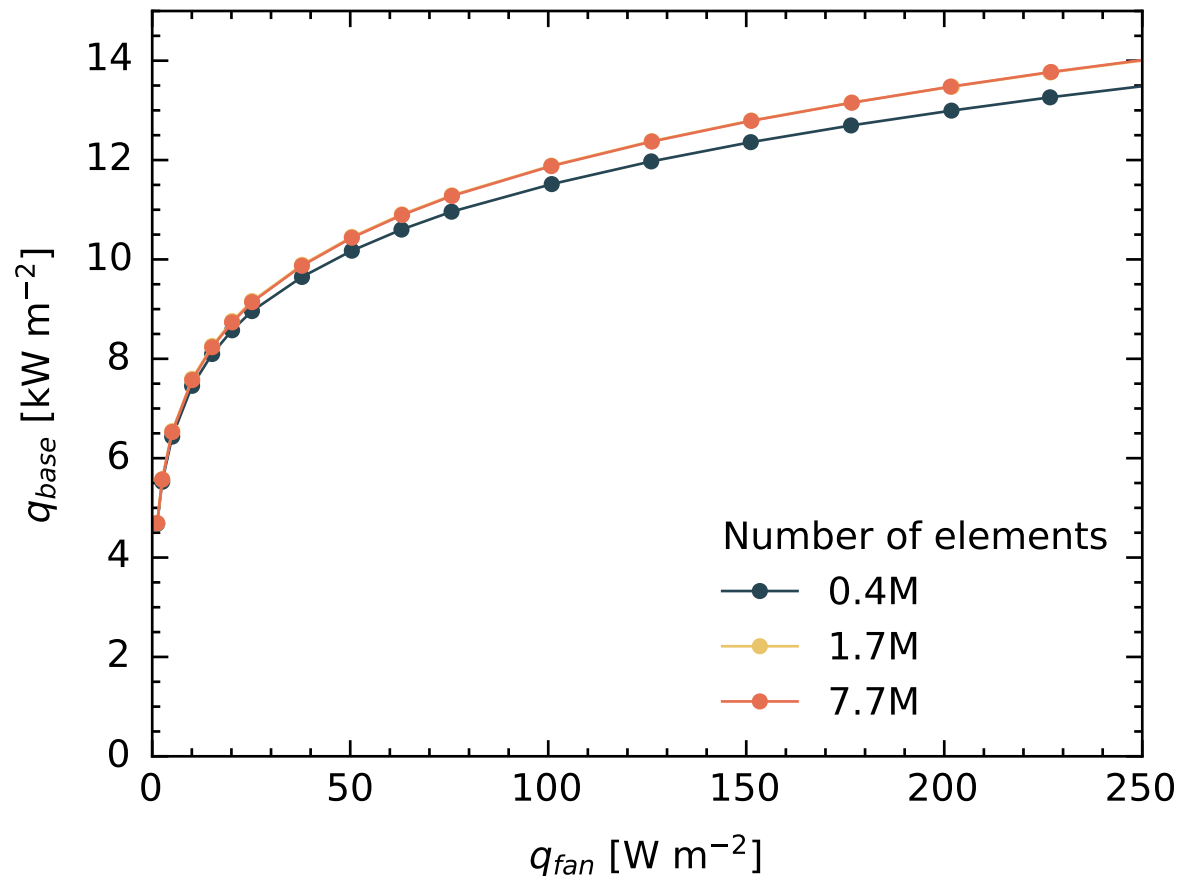

Fig. S5: Mesh-convergence analysis for the baseline HOMeS pin-fin geometry. The dissipated base heat flux q_{base} is plotted as a function of the fan power density q_{fan} for three mesh resolutions of 0.4, 1.7, and 7.7×10^6 control volumes (shown as dark teal, gold, and orange curves with filled circular markers, respectively; the 1.7 and 7.7×10^6 curves overlap). The selected mesh of $\sim 1.7 \times 10^6$ control volumes yields less than 0.5% maximum relative error over the full fan power density range compared to the finest mesh considered.

Note S6. Robustness of the area enhancement to fan power

The $\sim 7\times$ enhancement of the HOMeS area-enhanced channel over the flat evaporating channel is reported at the baseline fan power density $q_{\mathrm{fan}} = 125\ \mathrm{Wm^{-2}}$ in the main text. To confirm that this benefit is not specific to that operating point, the enhancement factor $q_{\mathrm{base,AE}}/q_{\mathrm{base,flat}}$ of the baseline HOMeS pin-fin channel (Fig. 2) relative to the flat evaporating channel is plotted against q_{fan} in Fig. S6, at the default inlet condition ($T_{\mathrm{in}} = 25\ °\mathrm{C}$, $\phi_{\mathrm{in}} = 50\%$). The ratio varies only weakly across the full fan-power range, rising from about 6.2 at $q_{\mathrm{fan}} = 1.25\ \mathrm{Wm^{-2}}$ to about 7.3 at 300 $\mathrm{Wm^{-2}}$ and reaching ≈ 7 already near $q_{\mathrm{fan}} = 40\ \mathrm{Wm^{-2}}$. In the low-fan-power regime of 1 to 2 W per panel, which corresponds to 25 to 50 $\mathrm{Wm^{-2}}$ for the 0.2×0.2 m footprint, the enhancement is ≈ 6.9 to $7.0\times$, essentially the same as at 125 $\mathrm{Wm^{-2}}$. The area-enhancement benefit therefore holds across the fan-power range and is not an artefact of the baseline operating point. The slight increase of the ratio with q_{fan} reflects the stronger boundary-layer disruption by the pin fins at higher flow rates, consistent with the macrochannel-height analysis in the main text.

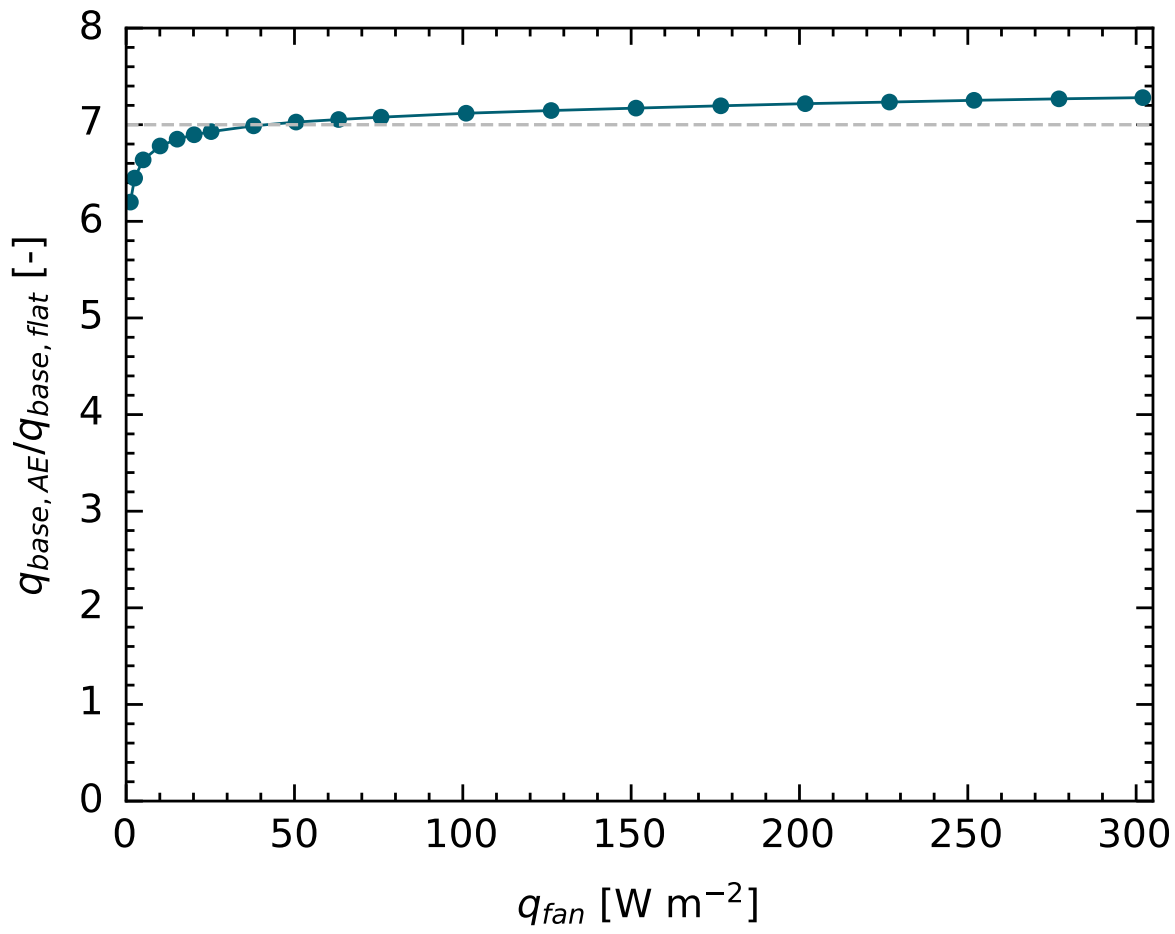

Fig. S6: Robustness of the area-enhancement factor to fan power. The enhancement factor $q_{\mathrm{base,AE}}/q_{\mathrm{base,flat}}$ of the baseline HOMeS area-enhanced pin-fin channel relative to the flat evaporating channel is plotted as a function of fan power density q_{fan} (solid dark teal curve with filled circular markers), at the default inlet condition ($T_{\mathrm{in}} = 25\ °\mathrm{C}$, $\phi_{\mathrm{in}} = 50\%$). The horizontal grey dashed line marks $7\times$. The enhancement rises from about 6.2 at the lowest fan power density to about 7.3 at the highest and remains $\approx 7\times$ in the low-fan-power regime ($q_{\mathrm{fan}} \lesssim 50\ \mathrm{Wm^{-2}}$).

Supplementary Figures

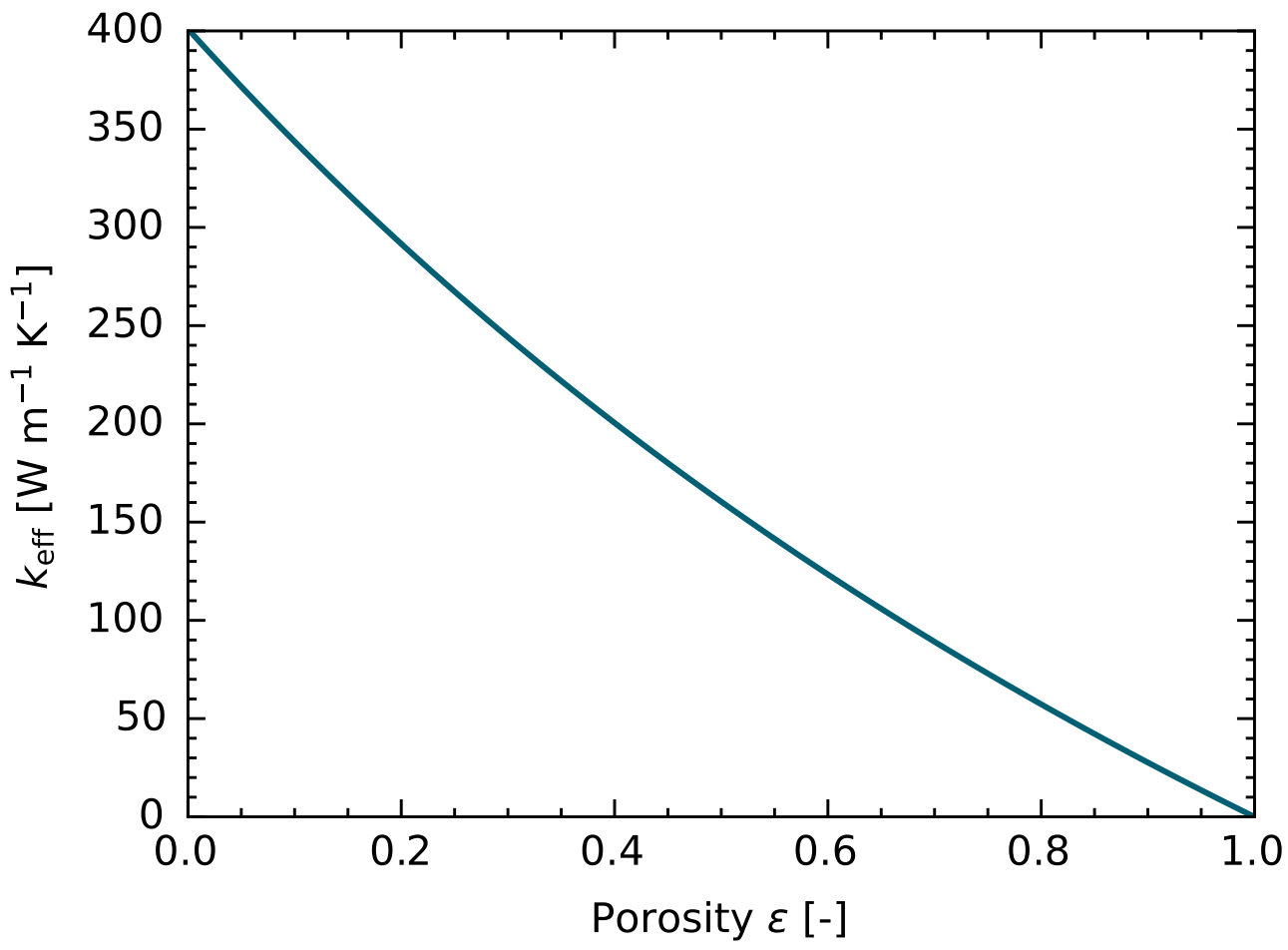

Fig. S7: Effective thermal conductivity of the liquid-saturated porous metal scaffold. The effective conductivity k_{eff} of a liquid-saturated porous copper scaffold, predicted by the Maxwell–Eucken model [2], is shown as a function of porosity ε (pore-filling phase: liquid water, $k_f \approx 0.6$ Wm^{-1}K^{-1}).

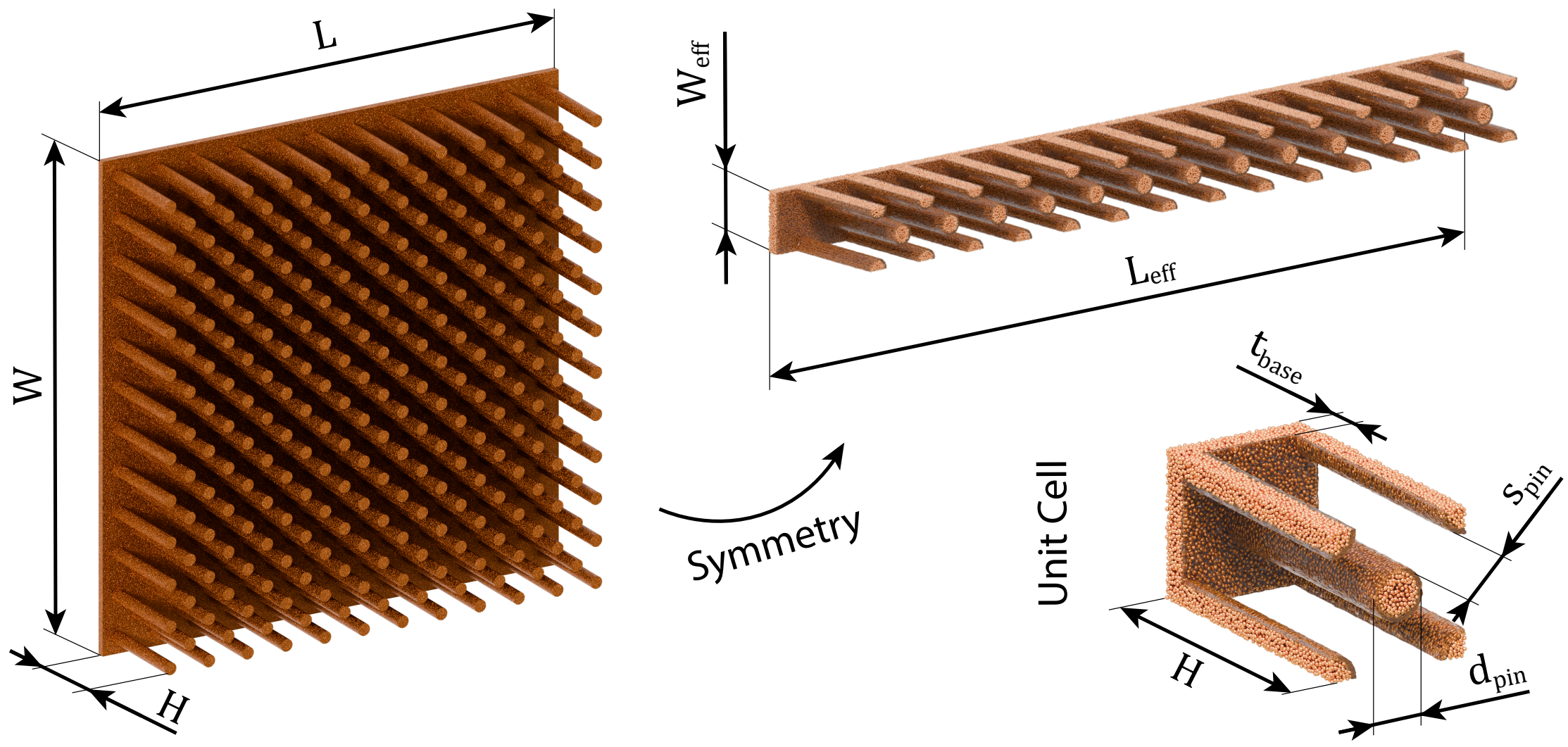

Fig. S8: Reduction of the computational domain to a single longitudinal pin-fin row. The macrochannel geometry (W, L, H) is reduced to a single longitudinal row by exploiting symmetry of the rotated square pin-fin lattice.

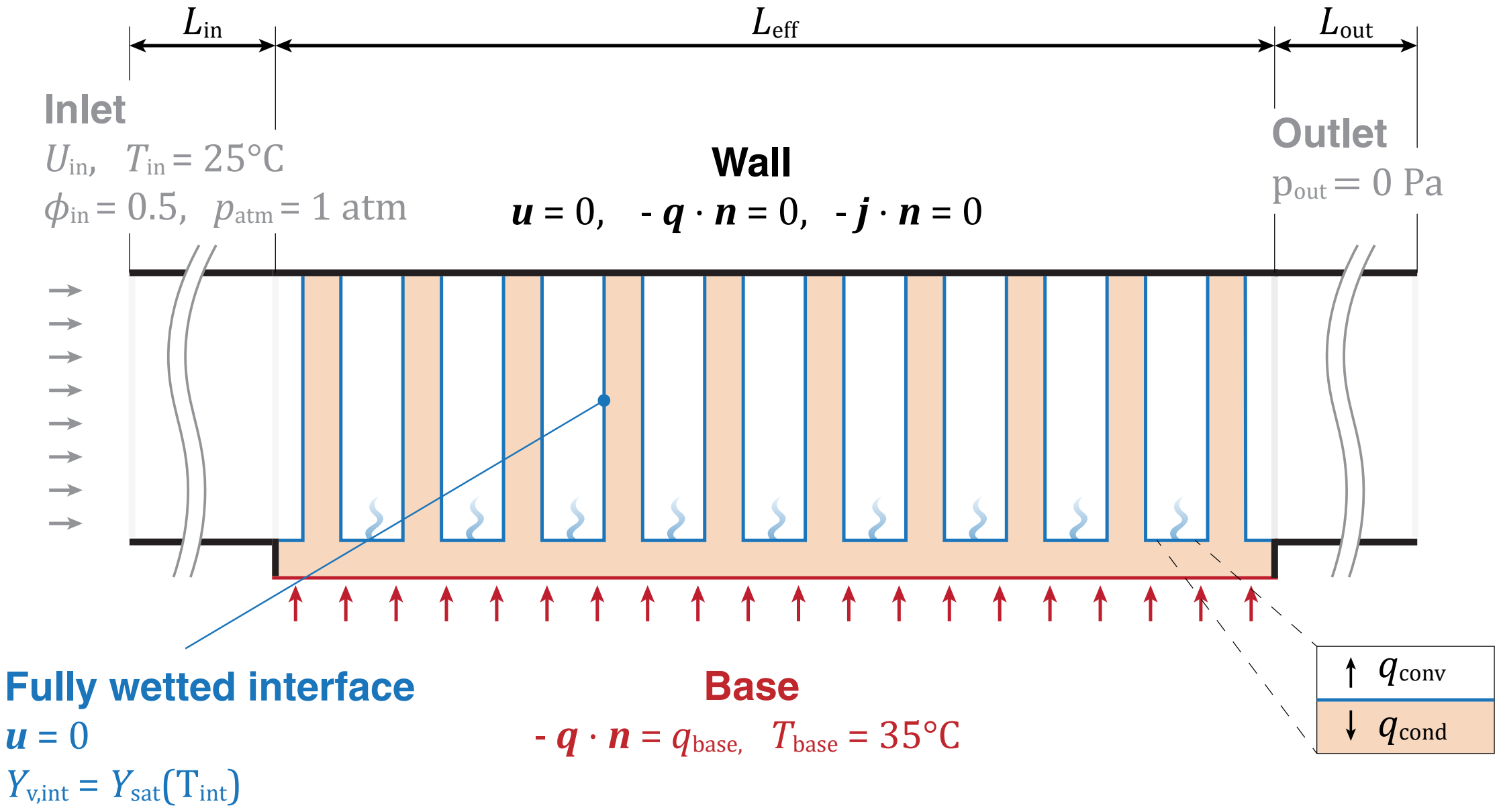

Fig. S9: Boundary conditions for the conjugate heat-mass transfer simulations. Humid air enters with prescribed U_{in}, T_{in}, and ϕ_{in} at 1 atm and exits at zero gauge pressure; entrance and exit extensions (L_{in}, L_{out}) are appended to the active section (L_{eff}). The base is held isothermal at T_{base}, the opposing wall is no-slip, adiabatic, and impermeable, and the fully wetted interface enforces $Y_{v,\text{int}} = Y_{\text{sat}}(T_{\text{int}})$ to couple vapor diffusion and latent heat transfer. The pressure drop $\Delta p_{\text{section}}$ is evaluated across the active section only.

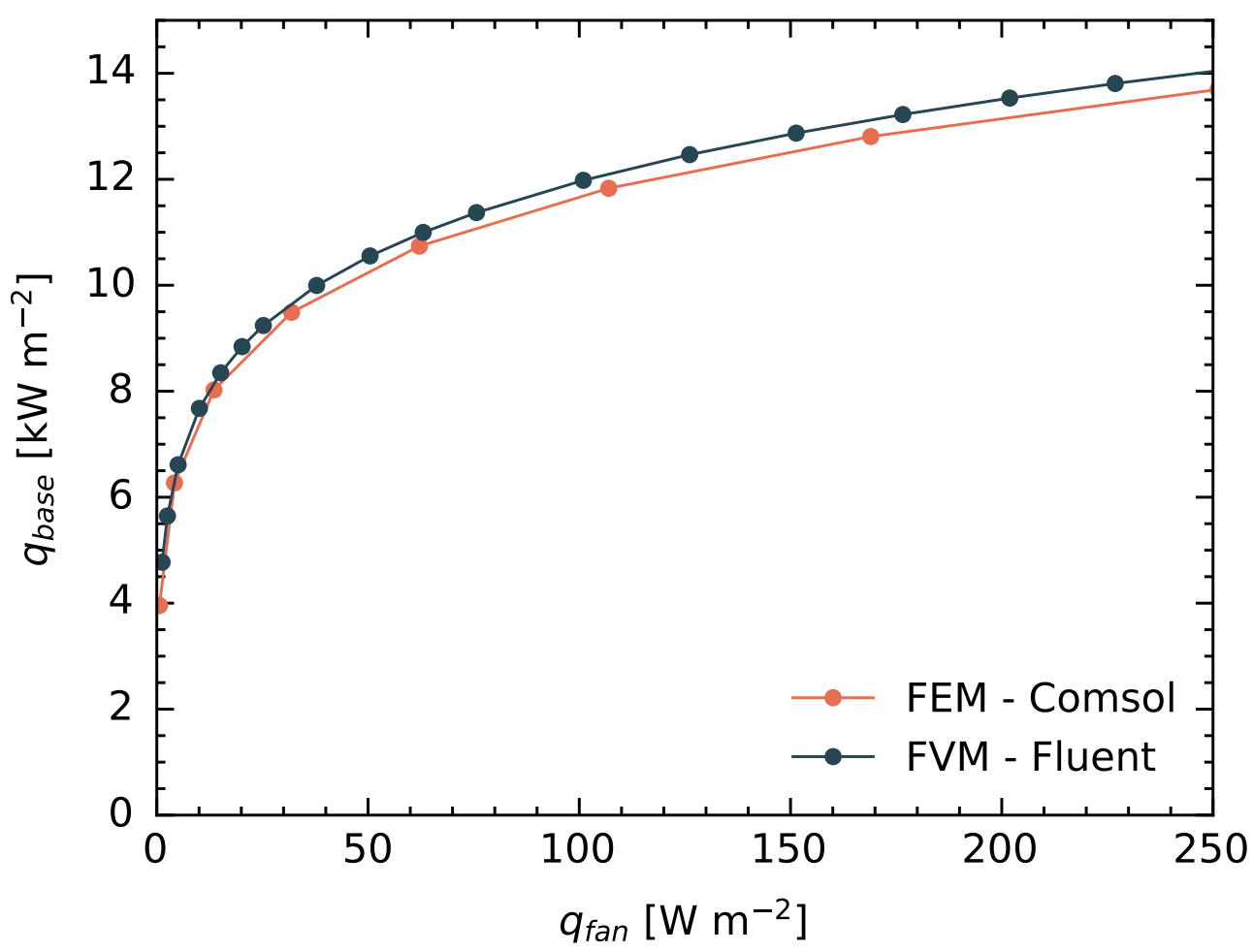

Fig. S10: Cross-verification of the finite-volume FLUENT solver against an independent finite-element implementation (COMSOL Multiphysics) for the baseline area-enhanced HOMeS configuration. The base heat flux q_{base} as a function of the fan power density q_{fan} obtained with the two solvers, shown as separate coloured curves as labelled in the legend, agrees closely, supporting the consistency of the CFD predictions.

References

[1] Carman, P. C. *Flow of Gases Through Porous Media* (Butterworths Scientific Publications, London, 1956).

[2] Wang, J., Carson, J. K., North, M. F. & Cleland, D. J. A new approach to modelling the effective thermal conductivity of heterogeneous materials. *International Journal of Heat and Mass Transfer* **49**, 3075–3083 (2006).
